# Preservation Strategies for Camel Meat: Quality Improvement and Shelf‐Life Extension

**DOI:** 10.1002/fsn3.71730

**Published:** 2026-04-07

**Authors:** Najmeh Rezaei, Farnaz Rezaiyan Attar, Nasser Morgan Azghadi, Mohammad Ali Hesarinejad

**Affiliations:** ^1^ Department of Food Hygiene, Faculty of Veterinary Medicine Semnan University Semnan Iran; ^2^ Central Laboratory of the General Directorate of Veterinary Affairs of Khorasan Razavi Province Iran Veterinary Organization Mashhad Iran; ^3^ Department of Food Sensory and Cognitive Science Research Institute of Food Science and Technology (RIFST) Mashhad Iran

**Keywords:** biopreservation, camel meat, irradiation, modified atmosphere packaging, nanotechnology, natural preservatives, thermal processing, traditional preservation, vacuum packaging

## Abstract

Camel meat, a valuable source of animal protein, plays a crucial role in food security, especially in arid and semi‐arid regions. However, its high moisture content, elevated pH, and low intramuscular fat make it highly susceptible to microbial spoilage and physicochemical changes, limiting its shelf life. This review provides a comprehensive overview of preservation techniques applied to camel meat. Traditional methods such as salting, drying, and smoking have been used for centuries to extend shelf life and improve sensory attributes. In contrast, modern approaches like refrigeration, freezing, vacuum packaging, and modified atmosphere packaging offer enhanced quality retention and microbial safety. Additionally, natural preservatives such as plant extracts, essential oils, and organic acids have gained popularity as clean‐label alternatives to synthetic additives like nitrites and potassium sorbate. Emerging technologies, including irradiation, nanotechnology, and biopreservation using beneficial microorganisms, show significant potential for improving the safety, nutritional value, and marketability of camel meat. Although substantial progress has been made, further research is needed to optimize these innovative methods and integrate them into industrial‐scale applications. Future developments that focus on eco‐friendly, minimally processed, and consumer‐accepted preservation strategies have the potential to significantly improve camel meat production and expand its global market presence. This review uniquely integrates traditional knowledge with cutting‐edge food technologies, offering a critical assessment of preservation methods specifically optimized for the distinct physicochemical properties of camel meat.

## Graphical abstract

Camel meat, a crucial protein source in arid regions, faces challenges like high moisture content and susceptibility to spoilage, limiting its shelf life. Traditional methods such as salting, drying, and smoking have been used historically, while modern techniques like refrigeration, freezing, and vacuum packaging enhance quality and safety. Natural preservatives from plants and essential oils offer clean‐label alternatives to synthetic additives. Emerging technologies, including irradiation and biopreservation, hold promise for improving safety and nutrition. Future efforts should focus on optimizing these methods for industrial‐scale applications to enhance the market presence of camel meat.

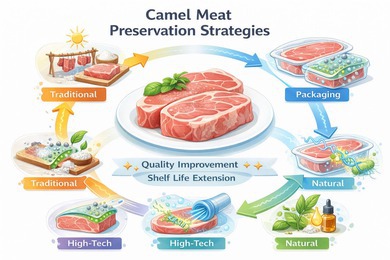

## 1 Introduction

Meat is a vital source of energy and nutrients, providing essential proteins, fatty acids, iron, vitamins, and minerals that significantly impact human health. The presence of these unique compounds has made meat and its products one of the most complete and widely consumed foods in the human diet (Jiang and Xiong 2016). Food preservation is defined as the process of treating and handling food to stop or greatly slow down spoilage and prevent foodborne illness while maintaining nutritional value, texture, and flavor. The primary goal of preservation methods is to extend the shelf life of food products by controlling or inactivating spoilage microorganisms and endogenous enzymes. These methods act through various mechanisms, including lowering temperature, reducing water activity, modifying the atmosphere, or adding antimicrobial agents. By creating an unfavorable environment for microbial growth and chemical reactions, preservation strategies ensure the safety and quality of meat products from the point of production to consumption.

Camel meat is at a similar level in nutritional value to other major sources of red and white meat, and may have lower intramuscular fat and cholesterol than beef or lamb (Elgasim and Alkanhal 1992). A distinctive feature of the dromedary camel ( *Camelus dromedarius* ) is its high adaptability to harsh environmental conditions, which strengthens its position as a valuable source of meat. Studies have shown that camel meat is similar to beef in terms of protein and moisture content, but has lower fat and ash (Reza Gheisari et al. 2009). Comparative analyses indicate that while camel and fish meat exhibit higher moisture content than beef, mutton, goat, and chicken, camel meat possesses slightly lower protein levels and the lowest ash content among these sources; furthermore, regarding fat and mineral composition, camel meat closely resembles beef but contains less fat and higher moisture (Elgasim and Alkanhal 1992; El‐Faer et al. 1991). The quality of camel meat depends on several factors such as age, sex, and nutritional status. Meat from younger camels (under 3 years of age) is comparable to beef in taste and texture, but camels are often slaughtered at advanced age, resulting in tougher meat (Alla 2008).

Camel meat contains significant amounts of essential amino acids, including lysine and methionine, and minerals, vitamins, and bioactive compounds such as carnosine, anserine, glutathione, and essential fatty acids such as omega‐3. Specifically, it provides approximately 3.5 to 4.0 mg of iron per 100 g, which is notably higher than beef (2.6 mg/100 g) and contributes effectively to preventing anemia (Kadim et al. 2014). Some other studies reported that camel meat contains lower levels of fat compared to other types of red meat, which could make it a healthier alternative for people looking to reduce their saturated fat intake. Furthermore, the fatty acids in camel meat have been linked to potential health benefits such as reduced inflammation and improved cardiovascular health (Reza Gheisari et al. 2009). Overall, studies suggest that camel meat can have significant health benefits and should be considered as a valuable addition to human nutrition. It is important to mention that, due to the high final pH of camel muscles, which is a result of low glycogen due to pre‐slaughter stresses, the microbial load of camel meat is sometimes higher than other meat sources. Therefore, keeping camel meat at refrigerated temperature is necessary to prevent the growth of pathogenic bacteria and spoilage agents, but it does not play a role in its long‐term storage (Eskandari et al. 2013).

Epidemiological and consumer studies have shown that food consumption patterns have changed worldwide and consumers have a great desire to use fresh food without preservatives (Kadim et al. 2014). However, like other sources of meat, camel meat is also subject to microbial spoilage. Camel meat spoilage can be caused by various microorganisms such as bacteria, yeasts, and molds. According to Odeyemi et al. (2020), bacterial spoilage of camel meat is primarily caused by Gram‐negative bacteria such as *Pseudomonas aeruginosa* , *Escherichia coli* , and LAB. These bacteria can multiply rapidly, with generation times as short as 20–30 min under optimal conditions, specifically at temperatures above 10°C, *a*_w_ greater than 0.95, and in the presence of oxygen. Similarly, another study reported that Enterobacteriaceae, lactic acid bacteria, and Bacillus species were the dominant bacteria responsible for the spoilage of camel meat. Yeasts and molds also cause spoilage of camel meat (Al‐Ani and Roberson 2005).

Several factors affect the shelf life and quality of meat; including storage temperature, atmospheric oxygen (O_2_), endogenous enzymes, moisture, light, and most importantly, microorganisms. Each of these factors, alone or in combination, can cause undesirable changes in the color, odor, texture, and flavor of meat. Although meat spoilage can also occur in the absence of microorganisms (e.g., proteolysis, lipolysis, and oxidation), microbial growth is the most important factor associated with the shelf life of fresh meat (Lambert et al. 1991).

Traditionally, meat preservation methods can be divided into three broad categories: temperature control, humidity control, and direct control with barrier processes (bactericidal and bacteriostatic, such as ionizing radiation, packaging, etc.). However, a preservation method can incorporate several antimicrobial principles simultaneously. Each control step can be considered as a “hurdle” against microbial proliferation, and combinations of methods (so‐called hurdle technology or HT) can be designed to achieve specific goals in terms of microbial and sensory quality (Lawrie and Ledward 2006).

**FIGURE 1:**
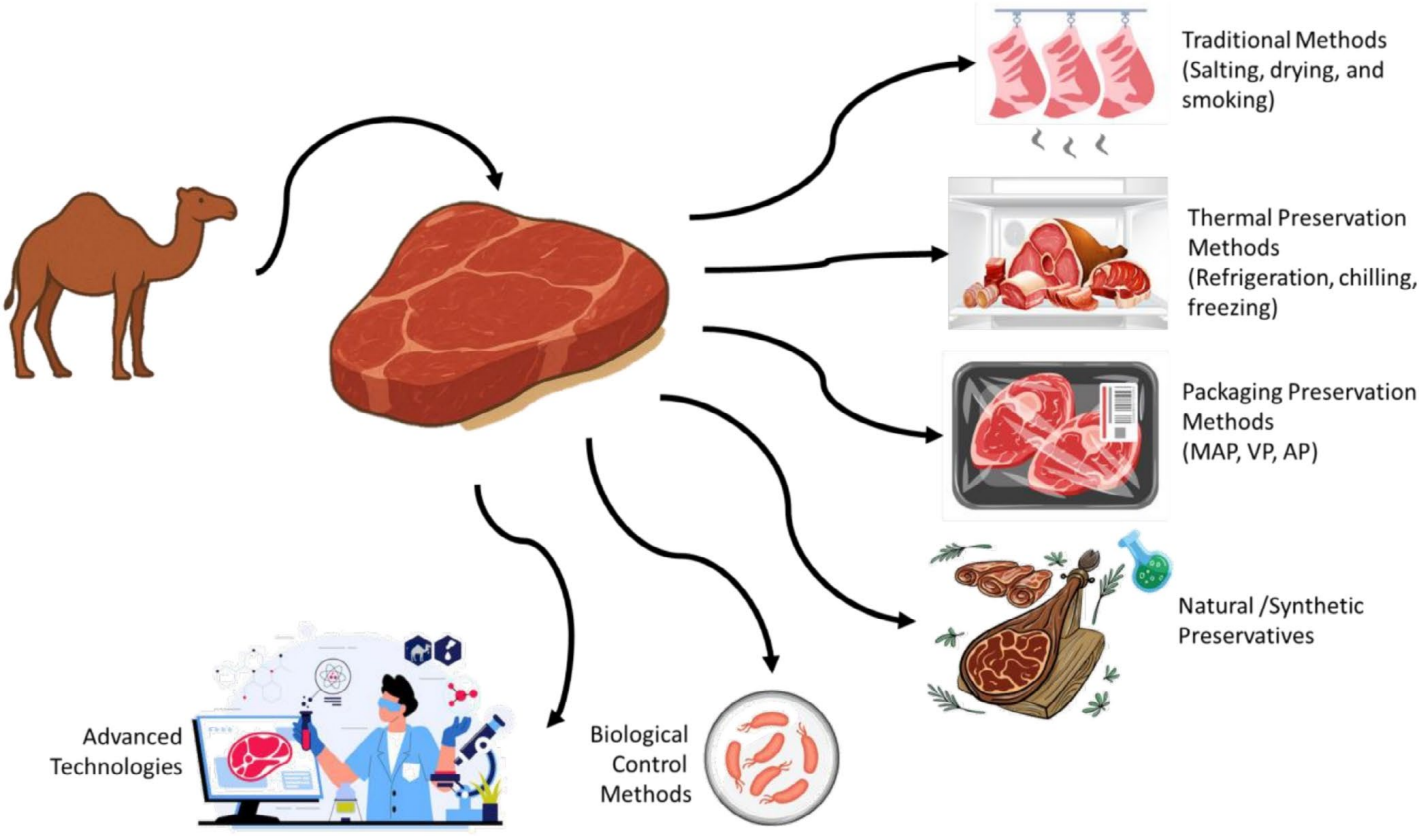
Preservation techniques to improve quality and extend shelf life of camel meat (assets from freepik.com were used).

The most investigated emerging non‐thermal methods such as modified‐atmosphere and active packaging, natural antimicrobial compounds, and biopreservation. All of these alternative technologies are designed to be gentle, energy‐efficient, environmentally friendly, and maintain natural appearance while eliminating pathogens and spoilage microorganisms. This review summarizes preservation strategies for fresh camel meat and their effects on quality and shelf life (Figure 1).

Although several reviews have addressed camel meat nutrition and general quality, there is a scarcity of comprehensive literature that consolidates preservation strategies specifically within the context of modern food safety challenges and consumer trends. Existing reviews often focus on traditional methods or nutritional aspects in isolation. The novelty of this review lies in its critical synthesis of both traditional and emerging non‐thermal technologies such as nanotechnology, biopreservation, and active packaging applied specifically to camel meat. Furthermore, this work uniquely discusses the limitations of current research and proposes future directions for industrial scalability, providing a holistic roadmap for researchers and industry stakeholders aiming to extend the shelf life of this valuable protein source.

## 2 Chemical Composition of Camel Meat

To understand the specific preservation needs of camel meat, it is essential to first examine its unique chemical composition. The intrinsic properties, such as moisture content, pH, and fat profile, dictate how the meat responds to spoilage mechanisms and preservation technologies. Therefore, the following section details the proximate composition of camel meat (Table 1).

**TABLE 1:** Comparison of chemical compositions of various meat sources (McCance and Widdowson 2014; Kadim et al. 2006).

Meat source	Moisture (%)	Protein (%)	Fat (%)	Energy (kJ/100 g)
Camel	70–76	17–23	1.4–5	450–650
Beef	55–72	18–24	2–15	500–900
Sheep	55–70	16–22	8–25	600–1100
Chicken	65–75	19–23	1–10	400–800
Turkey	65–74	19–24	1–10	400–750
Fish	60–80	16–22	0.5–15	300–900

Fresh camel meat typically has a moisture content ranging from 70% to 73% which may vary depending on factors such as the age, sex, and diet of the animal (Babiker and Yousif 1990). The water content of processed camel meat products such as sausages can vary greatly based on the processing method used. For example, some studies have reported water content ranging from 10% to 40% in camel meat sausages on a dry basis, influenced by formulation and drying process (Zaki 2017).

Regarding protein, it has been reported a mean protein content of 21.8% ± 1.2% on a wet weight basis, which is comparable to standard beef values (Babiker and Yousif 1990). Kadim et al. (2022) noted that protein levels generally range between 17% and 23%, varying by meat cut and factors such as breed, age, diet, and processing methods (Kadim et al. 2022).

Babiker and Yousif (1990) reported that camel meat is generally lean, with a fat content ranging from 1.5% to 4%, depending on the cut of meat and the age of the animal (Babiker and Yousif 1990). Reza Gheisari et al. (2009) reported that camel meat contains lower amounts of saturated fat and higher amounts of unsaturated fatty acids compared to other types of meat, making it a healthier option. These studies show that camel meat is generally lower in fat than other red meats such as beef or pork, although the specific fat content may vary depending on factors such as the cut of meat and the age of the animal (Reza Gheisari et al. 2009).

According to a study by Hamad and Abdullah (2025), the carbohydrate content of camel meat was low compared to beef and lamb. This study showed that camel meat had an average carbohydrate content of 0.38%, while beef and lamb had average values of 1.6% and 1.8%, respectively (Hamad and Abdullah 2025).

Several studies have examined the vitamin and mineral content of camel meat. Raiymbek et al. (2025) reported that camel meat is a good source of thiamine, riboflavin, pantothenic acid (vitamin B5), vitamin B6, and vitamin B12 (Raiymbek et al. 2025). In addition, this study showed that it is also contains appreciable amounts of iron, zinc, and phosphorus. In another study, Kadim et al. (2006) found that camel meat is a rich source of important minerals such as calcium, magnesium, potassium, and sodium (Kadim et al. 2006).

Although the nutritional profile of camel meat is advantageous, the chemical components discussed above also render it susceptible to specific deterioration pathways. The high moisture content and specific lipid profile create a conducive environment for spoilage agents. Consequently, understanding the factors that accelerate these degradation processes is a prerequisite for selecting effective preservation strategies, as discussed in the next section.

## 3 Factors Affecting Camel Meat Spoilage

High water activity (*a*_w_) favors microbial growth; thus, greater *a*_w_ increases spoilage risk. On the other hand, proteins are degraded after slaughter by endogenous enzymes and microbial enzymes, which is associated with the production of spoilage products such as biogenic amines (Baba et al. 2021).

Camel meat is higher in polyunsaturated fatty acids (PUFA) than other meat sources. Unsaturated fatty acids are more susceptible to oxidation; that is, high PUFA levels can lead to oxidative spoilage (e.g., formation of TBARS and peroxides). Meat spoilage occurs when the free radicals produced in the first two steps interact with each other and form non‐radical products. Lipid oxidation is influenced by antioxidants (e.g., vitamin E) and fatty‐acid composition. The decomposition of hydroperoxides releases several products such as acids, ketones and aldehydes. This effect causes a decrease in nutritional value and loss of color, as it has an adversely affects nutrients and color stability and reduces nutritional quality and sensory acceptability. Lipid hydrolysis in meat can be carried out enzymatically or non‐enzymatically, and while enzymatic hydrolysis is carried out with the help of several enzymes such as phospholipase and lipase, the main enzymes involved in lipid hydrolysis in meat are phospholipase A1 and A2. Heme proteins such as hemoglobin, cytochrome, and myoglobin catalyze non‐enzymatic lipid oxidation (Shaltout et al. 2018, 2014, 2020; Edris et al. 2020; Sobhy and Shaltout 2020). In addition to chemical and enzymatic changes, microbial activity plays a pivotal role in the spoilage of camel meat. The chemical environment described in the previous section supports the growth of specific microorganisms that ultimately determine the shelf life. Thus, identifying the dominant microbial flora and their spoilage mechanisms is critical.

## 4 Microbial Flora and Microbial Spoilage of Camel Meat

The natural microbial flora of camel meat has been investigated in several studies. According to Tegegne et al. (2019), camel meat contains a diverse range of microorganisms including bacteria, yeasts, and molds. In addition, Enterobacteriaceae such as *Escherichia coli* and *Staphylococcus aureus* are the predominant species in freshly slaughtered camel meat samples. Other bacterial species identified included *E. coli* , *Pseudomonas*, *Salmonella* spp., and *Listeria* spp. (Tegegne et al. 2019). Furthermore, a study by Osaili et al. (2021) reported that the natural microbial flora of camel meat can be influenced by several factors, including the age of the animal, the type of feed, and the processing methods used. They found that younger animals had a greater diversity of microorganisms, and they also found that animals fed concentrates had a higher prevalence of potentially pathogenic bacteria. However, with increasing storage time, Enterobacteriaceae and *Pseudomonas* species became dominant, indicating a shift from LAB‐dominated bacteria to spoilage‐associated bacteria. Another study reported that Enterobacteriaceae, *Pseudomonas* species, and *Aeromonas* were the main bacteria responsible for spoilage of camel meat during refrigerated storage (Odeyemi et al. 2020). Consequently, the microbial flora of camel meat during storage and spoilage is affected by several factors, including storage temperature, packaging materials, and initial microbial load. Understanding and controlling these factors is crucial to ensure the safety and quality of camel meat (Osaili et al. 2021). Given the complex microbial ecology and the rapid spoilage potential of camel meat, effective intervention strategies are necessary. Historically, humans have relied on traditional methods to mitigate these challenges. These techniques, often passed down through generations, form the foundation of modern food preservation. The following section explores these traditional methods and their underlying principles.

## 5 Traditional Preservation Techniques

### 5.1 Salting and Drying

**FIGURE 2:**
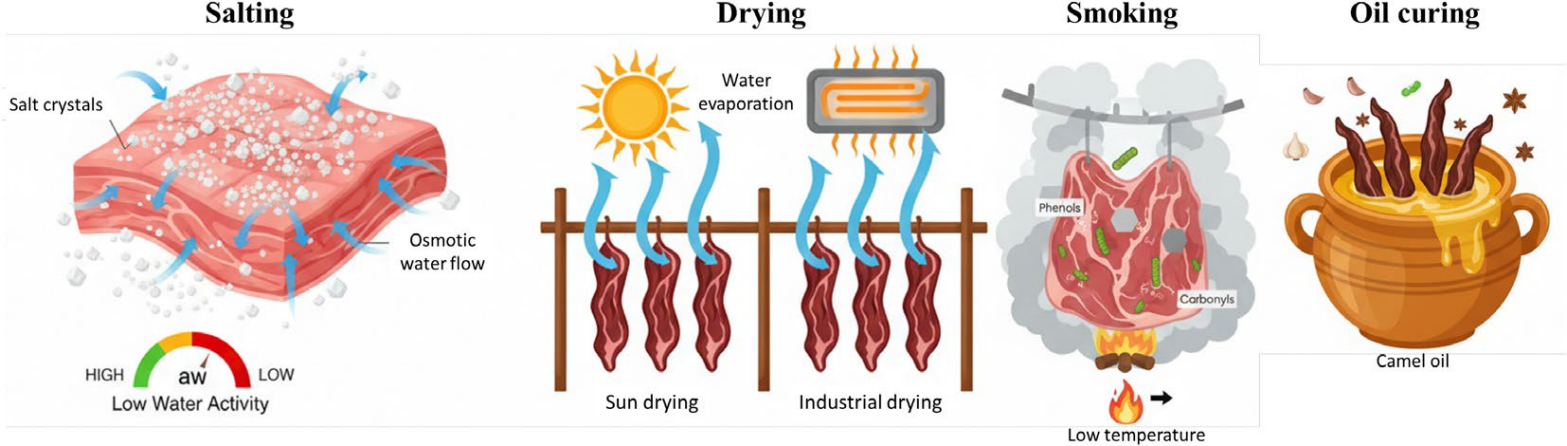
Traditional preservation of camel meat.

Camel meat has a limited shelf life due to spoilage. Traditional methods like sun drying and salting are used to extend its shelf life and prevent decay (Figure 2). Dried products produced by various methods (either open‐air drying or solar/industrial drying methods) are still of interest because they do not require a refrigeration system during distribution and storage. Drying technology is fundamentally based on reducing water activity and thus preserving the characteristics of the meat. Also, the use of salt reduces the water activity (*a*_w_) of camel meat and inhibits the growth of spoilage and pathogenic microorganisms. Benyagoub and Bessadet (2023) described the traditional Algerian product Kadid, produced by salting and sun‐drying camel meat slices. This process can safely extend shelf life for up to 6–12 months at ambient temperature (25°C–30°C) when stored in dry conditions (Benyagoub and Bessadet 2023). Some other studies also showed that fat oxidation is one of the main factors in the decline of the quality of camel meat products and can negatively affect the taste, color, and nutritional value of the product. A study on camel meat showed that the use of mixed curing processes can affect the activities of antioxidant enzymes such as catalase and glutathione peroxidase (GSH‐Px) and lipid oxidation indices. In this study, *Longissimus dorsi* samples (five adult males) were stored at 4°C for up to 12 days with different salt mixtures. The results showed that there was no significant difference in catalase activity and peroxide content between the control and treated groups, but GSH‐Px activity decreased during the storage period, and this decrease was greater in the control group. Also, the vitamin E content in the treated samples was higher and the thiobarbituric acid (TBA) content was lower than in the control group (Gheisari and Eskandari 2013). These findings indicated that the curing process can reduce fat oxidation and the resulting flavor deterioration and preserve meat nutrients such as vitamin E, thus improving the quality and shelf life of camel meat (Gheisari and Eskandari 2013).

In Algeria, camel meat processed, which is called Khlii, preserved in salt and spices can be stored in clay or glass containers. This mixture of dried and cooked meat lasts up to 3 years at room temperature if protected from sunlight and humidity (Benyagoub and Bessadet 2023).

Oil drying, known as the Moyale method in East Africa, is another traditional preservation technique (Figure 2). In Moyale, Kenya, camel meat is preserved as “hilib gel” sun‐dried strips dipped in camel oil, spiced with garlic, and then soaked in camel oil (gehai) to extend its shelf life by 4–6 months (Benyagoub et al. 2025).

### 5.2 Smoking

Smoking is one of the oldest methods of food preservation, used since humans began consuming meat. In this method, food is exposed to smoke from burning wood or other plant materials. This method helps preserve food to some extent, especially by drying the surface, which removes moisture from the meat, but it is not a reliable method of preservation alone unless combined with salting or drying (Busboom 2003). It also maintains tenderness when conducted at low temperatures (Figure 2). Smoking dries out the outer coating of the meat to a point where bacteria cannot enter. Smoking is often combined with salting for extended preservation. Common smoking methods include hot smoking, smoke roasting, and cold smoking. Hot smoking and roasting cook the meat, whereas cold smoking does not. When meat is processed by cold smoking, it should be dried quickly to limit bacterial growth, for example by making jerky or thinly sliced meat (Chellaiah et al. 2020). Care should also be taken to avoid direct wood smoke on the meat, as this can lead to the formation of carcinogenic polycyclic aromatic hydrocarbons in the food. Smoking camel meat introduces phenolic and carbonyl compounds with antimicrobial and antioxidant properties, and in addition to flavoring, it increases shelf life. According to Zegeye's research, 3‐h smoking with salt reduced *Pseudomonas* growth rate and could help improve the microbial safety of camel meat products (Zegeye 1999; Chellaiah et al. 2020).

Although traditional methods are effective, they often alter the sensory characteristics of the meat significantly. To maintain quality closer to the fresh state, modern thermal preservation methods focus on controlling temperature to slow down metabolic processes. The following section examines how low‐temperature storage can extend shelf life while preserving freshness.

## 6 Thermal Preservation Methods

### 6.1 Refrigeration

Low‐temperature storage slows microbiological, chemical, and enzymatic deterioration, helping to maintain freshness. Therefore, it is of particular importance to study and monitor the changes that meat experiences during low‐temperature storage. For example, post‐slaughter refrigeration, often referred to as “maturation,” induces favorable changes in the structure of myofibril proteins and connective tissue, which leads to improved meat palatability (Takahashi 1996). Meat carcasses are stored for various periods of time under refrigerated conditions to increase meat tenderness, which is its most important quality characteristic. In addition, temperatures below or above the optimal range for microbial growth have an inhibitory effect on it (Maqsood, Abushelaibi, Manheem, Al Rashedi, and Kadim 2015; Maqsood, Abushelaibi, Manheem, and Kadim 2015). Some researchers compared protein and lipid changes in camel, beef, and mutton during 9 days of refrigerated storage. Synthesizing these findings, camel meat is reported to be more susceptible to lipid oxidation than beef and mutton due to its high levels of unsaturated fatty acids, hemoglobin, and myoglobin. While fresh camel meat initially exhibited superior textural properties compared to the other meats, these attributes declined between days 3 and 9 due to proteolysis. Although refrigeration slows spoilage, it is insufficient for long‐term preservation (Maqsood, Abushelaibi, Manheem, and Kadim 2015; Manheem et al. 2023).

### 6.2 Chilling

Early societies recognized the role of low temperatures in increasing the shelf life of perishables, leading them to store meat and other products in natural caves at relatively constant and cool temperatures. The concept of artificial ice production and mechanical refrigeration systems dates back to the 18th century, around 1750, and nearly a century later, large‐scale commercial use of this technology began. The chilling process is now considered one of the most important methods for ensuring microbial safety, hygiene, increasing shelf life, and improving the appearance and sensory quality of meat (Lawrie and Ledward 2006). Reducing the surface temperature of the carcass and gradually drying it during air chilling creates conditions that limit bacterial growth. Controlling the intensity of the airflow and regulating the temperature can reduce chilling time, although rapid heat removal from the deep tissues of the carcass remains a serious challenge. There are different methods for chilling. In a natural convection system, where the refrigerant passes only through the cooling tubes, the process is slow and uncontrollable. In contrast, the use of forced convection combined with airflow generated by fans is more efficient. Rapid chilling of the carcass increases product yield while reducing surface evaporation and, due to rapid surface drying, also limits microbial growth. However, if this process is carried out before rigor occurs, it may lead to cold shortening and ultimately meat toughness. In addition, the use of a spray method during chilling can increase the oxygenation of surface myoglobin while preventing the formation of metmyoglobin; as a result, the desired color and initial weight of the meat are maintained (Feldhusen et al. 1995). Hassanien et al. (2022) aimed to evaluate the changes in beef and camel meat during the chilling process at 4°C and its role in the shelf life of these meat sources. The parameters studied included sensory characteristics (color, odor, consistency, and appearance), microbiological characteristics (total bacterial count, yeast and mold count, coliforms, and *Staphylococcus aureus* ), and chemical parameters (hydrogen potential (pH)), thiobarbituric acid (TBA), total volatile nitrogen (TVN), peroxide value (PV), glutathione peroxidase (GSHPx), catalase (CAT), amino acid composition, fatty acid composition, and free fatty acids (FFAs). Beef retained acceptable quality for 6–8 days, camel meat for up to 10 days. Chemically, the results for beef until the eighth day and for camel meat until the tenth day (for pH, TBA, TVN, PV, glutathione peroxidase, catalase and free fatty acids, as well as the fractionation of amino acids and fatty acids) were evaluated within acceptable limits. In summary, chilling storage at 4°C increased the shelf life of fresh camel meat by 8 days and fresh beef by 6 days, without having any adverse or detrimental effects on their sensory acceptance (Hassanien et al. 2022).

### 6.3 Freezing

Freezing is one of the common and effective methods of preserving meat, which increases shelf life and prevents microbial and chemical changes by reducing the temperature below the freezing point (Lawrie and Ledward 2006). The speed of freezing plays an important role in that rapid freezing prevents water loss during thawing by forming small ice crystals. A temperature of −55°C has been reported as effective for reducing enzymatic reactions and oxidation (Hansen et al. 2004). In addition to conventional freezing, the cryogenic method also shortens the freezing time by using liquid gases, but its main limitations are its high cost and the possibility of product deformation (Zhou et al. 2010). Reza Gheisari et al. (2009) compared the biochemical and functional properties of fresh and frozen camel meat to beef. The researchers randomly selected 24 camels and cows of varying ages and sexes and measured the chemical composition, pH, water holding capacity (WHC), total volatile nitrogen (TVN), peroxide value, acid value, tensile strength, and myofibrillar protein electrophoresis in meat samples. After freezing samples for 1, 4, and 8 weeks at −18°C and thawing, they assessed WHC, dripping loss, TVN, peroxide value, acid value, and Kreis test at each interval. The study concluded that freezing effectively delayed changes in all measured parameters of camel meat, and that camel meat's quality was comparable to, and potentially superior to, beef or sheep due to its lower intramuscular fat and cholesterol content (Reza Gheisari et al. 2009).

Temperature control alone is often insufficient for long‐term distribution, requiring supplementary barriers against environmental factors. Packaging technologies provide these necessary barriers, protecting meat from oxygen, moisture loss, and contamination. Therefore, modern packaging systems are integral to the preservation chain, as detailed below.

## 7 Packaging Preservation Methods

### 7.1 Vacuum Packaging

**FIGURE 3:**
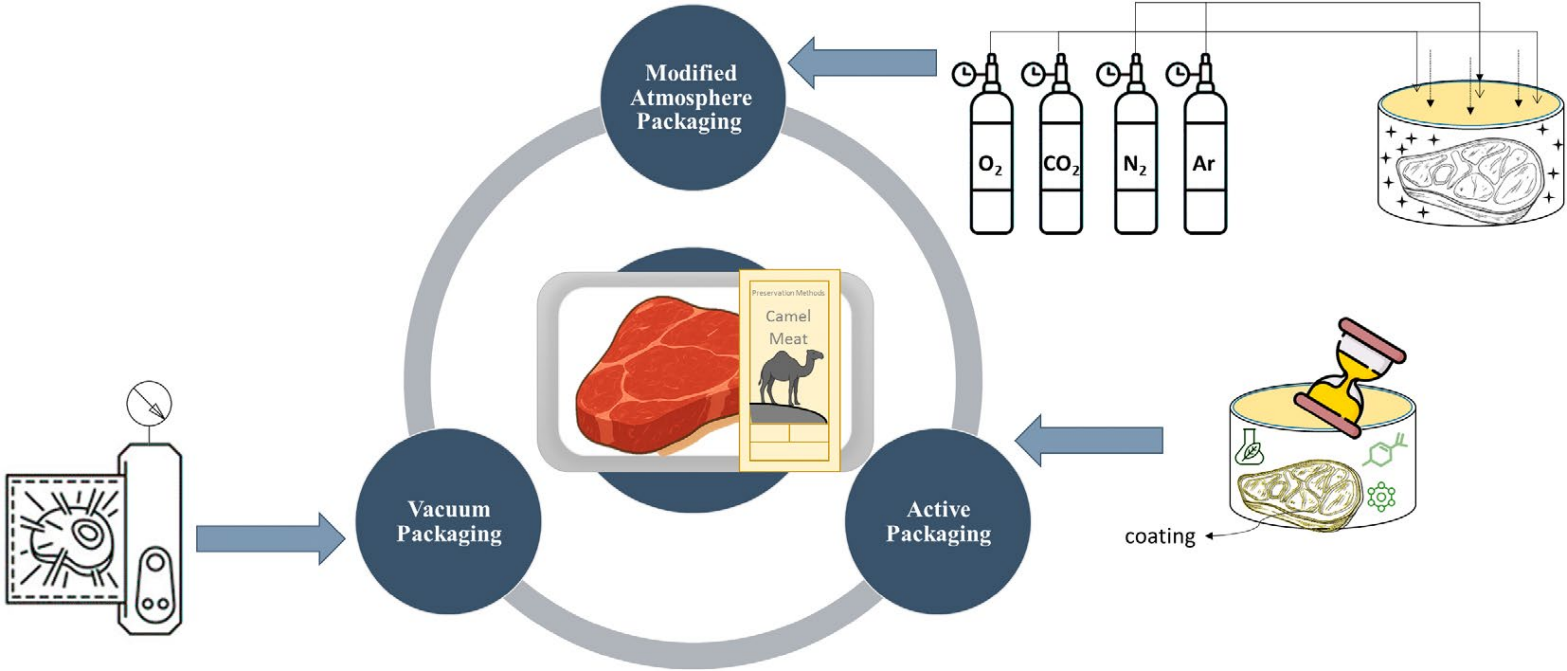
Type of Camel meat packaging preservation methods.

The use of appropriate packaging and storage conditions can play an important role in improving the color and preservation of meat during storage. Recent strategies by industry and researchers have been directed towards the use of packaging systems without synthetic additives, which can reduce fat oxidation and off‐odor formation, and significantly delay microbial growth. Vacuum packaging creates anaerobic conditions that increase both microbial and oxidative stability of meat (Maqsood et al. 2016). Removing oxygen from the packaging or using low‐permeability films inhibits the growth of obligate aerobic microorganisms and delays the growth of facultative microorganisms, as well as fat oxidation (Figure 3). Vacuum packaging can also facilitate transportation, enable more effective marketing, and extend the shelf life of the product (Table 2). Maqsood et al. (2016) investigated the effects of different packaging conditions, air, vacuum and coated packaging, on various quality attributes of camel meat during 18 days of refrigerated storage. The results showed that vacuum packaging reduced lipid oxidation rates by approximately 40% compared to air packaging, as measured by Thiobarbituric Acid Reactive Substances (TBARS) values after 18 days of storage. In addition, sensory evaluators rated the quality of camel meat packaged in vacuum packaging superior to meat packaged under other packaging conditions (Maqsood et al. 2016). Therefore, vacuum packaging can maintain the quality of camel meat for a longer period by limiting microbial load, lipid oxidation and maintaining meat color. Therefore, it can be used as an effective strategy in the storage and distribution of fresh camel meat in refrigerated shelves of stores as well as in international transportation without the need for freezing.

**TABLE 2:** Packaging methods applied to camel meat products.

Packaging type	Gas/treatment	Conditions	Outcome	References
Vacuum packaging	Anaerobic (no oxygen)	18 days at 4°C	Delayed lipid oxidation and microbial growth, maintained redness	Maqsood et al. (2016)
MAP	60% CO_2_ + 40% N_2_	21 days at 4°C	Extended shelf life to 21 days, improved color and sensory quality	Jouki and Khazaei (2012)
MAP (high O_2_)	80% O_2_ + 20% CO_2_	30 days at 1°C ± 1°C	Reduced TBARS and Pseudomonas growth, increased shelf life	Djenane et al. (2020)
Active packaging (Chitosan + Citrox)	1.2% Citrox +1% chitosan	Vaccum Packaging, 30 days at 4°C and 10°C	Inhibited *C. jejuni* , shelf life > 30 days	Yehia et al. (2021)
Active packaging (Arabic gum)	10% Arabic gum	14 days at 4°C	Inhibited *Klebsiella pneumoniae* , shelf life increased to 14 days	Elsharawy et al. (2026)
Active packaging Lactic acid (1.7%) and acetic acid (2%)	Edible coatings on meat	4°C	Reduced microbial count by 1.5 and 0.25 log units, respectively	Siragusa and Dickson (1992)
Nanocomposite films (MMT‐Ch, MMT‐CMC + essential oil)	0.5%–2% Ziziphora oil + fig extract	Cold storage	Reduced *L. monocytogenes* and *E. coli* , improved quality, Reduced TBARS, TVN, PV	Khezrian and Shahbazi (2018)
Bioactive basil seed gum film (resveratrol +clove oil)	Nanoemulsion‐based film	20 days at 4°C	Improved oxidative stability, better sensory quality	Ansarian et al. (2022)

### 7.2 Modified Atmosphere Packaging (MAP)

Modified atmosphere packaging of meat requires the use of materials that prevent the ingress of moisture and gases to maintain a stable environment during the storage period (Figure 3). In each type of MAP, the natural composition of the atmospheric air must be removed or altered. This technology includes both aerobic and anaerobic packaging of meat. The main gases present in dry air at atmosphere by volume are nitrogen (78%), oxygen (20.99%), argon (0.94%), and carbon dioxide (0.03%), although the percentages vary (McMillin 2008). Although low‐oxygen MAP (Low O_2_ MAP) is available, it is less commonly used, while vacuum packaging remains the most cost‐effective packaging method (McMillin 2008). Low‐oxygen MAP packaging can use an anaerobic atmosphere consisting of nitrogen and carbon dioxide. Nitrogen is a neutral gas that does not react with meat pigments and is not absorbed by the meat; therefore, its presence maintains the stability of the packaging environment. In contrast, carbon dioxide reacts with the meat and changes its properties. In this method, barrier trays are filled with the product and then sealed with a barrier lidding film after injecting the desired gas composition. These trays are usually manufactured ready‐made. This process causes the meat pigment to be in the deoxymyoglobin state, which appears purple; a color that may be unfamiliar to many consumers (Jenkins and Harrington 1991). Non‐barrier‐coated meat packages can be placed in barrier bags that are either individually packaged (tray‐in‐sleeve configuration) or as group packs within a larger barrier film (master pack) with anaerobic gas. In this case, the meat pigments are oxygenated and turn red when the package is removed from the permeable film and exposed to air (McMillin et al. 1999). Another method of using anaerobic MAP is with multilayer films: an inner layer that is air‐permeable and an outer layer that is a barrier. When the outer film is removed before release to the market, the meat is exposed to oxygen in the air and its color improves (blooming). In cases where permeable films do not allow sufficient oxygen to pass through to form oxymyoglobin, microporous shrink films with additional holes or pores have been developed to allow the reddening process to occur more rapidly (Zhou et al. 2010).

Carbon monoxide (CO) has also been used in low oxygen MAP packaging. The meat may be exposed to CO before packaging or CO may be used to fill the vacuum space packaging (VSP). Even small amounts of CO are sufficient to produce the desired red color in the meat. However, the most common type of MAP for fresh meat is high oxygen packaging (about 80%) O_2_, which provides sufficient shelf life for producers and retailers in controlled distribution systems (Zhou et al. 2010). A study by Jouki and Khazaei (2012) investigated the effect of lipid oxidation on the color and sensory properties of fresh camel meat stored at 4°C under modified atmosphere packaging conditions (AP: air packaging, VP: vacuum packaging, MAP: 60% CO_2_ + 40% N_2_). The color results showed that MAP performed best among the tested methods. The study also showed that although the lipid oxidation index in air‐packed samples increased with time, this increase did not lead to a decrease in sensory quality until the 14th day. The sensory evaluation results were generally consistent with the physicochemical changes and showed that MAP has a significant effect on the quality of refrigerated camel meat. Packaging of fresh camel meat under modified atmosphere combined with storage at refrigerated temperature increased the shelf life of the product by up to 21 days without causing adverse effects or reducing sensory acceptance (Jouki and Khazaei 2012). In a study by Djenane et al. (2020), the effect of combined treatments on the quality characteristics of camel steaks packaged under modified atmosphere conditions with high concentrations of O_2_ (80%) and CO_2_ (20%) during long‐term storage at refrigerated temperature (1°C ± 1°C) was investigated. Treated camel steaks had lower concentrations of thiobarbituric acid‐releasing compounds (TBA‐RSs) during 30 days of storage. The amount of surface metmyoglobin (MetMb) in the treated samples increased at a lower rate than in the control samples. After 30 days of storage, the number of psychrotrophic bacteria and *Pseudomonas* spp. in camel steaks was significantly lower than that in untreated samples. Overall, MAP is a sustainable and economical technology for meat packaging industries that can provide more opportunities for market development and increase the exportability of camel meat and other meat products while ensuring microbiological safety, chemical stability, and sensory quality (Djenane et al. 2020).

### 7.3 Active Packaging

Recent advances in packaging technology have been made to improve the physical, chemical, microbial, and organoleptic properties of food. Conventional or passive packaging is made from materials such as plastic, glass, and wood that create an impermeable layer on the food, while active packaging, as another type of packaging, is made from natural or synthetic compounds and is used to increase the shelf life of food or improve the properties of the coating. Antimicrobial packaging is one type of active packaging that controls chemical spoilage, pathogens, and spoilage agents by incorporating antimicrobial and antioxidant materials into the coating or packaging (Kerry et al. 2006). For optimal use of antimicrobial materials, factors such as the effectiveness of the film and the added antimicrobial compounds against the target bacteria, the mechanism of release of the effective compounds from the packaging into the food, and the interaction between the film and the food must be considered (Figure 3). For example, Chitosan is a natural polysaccharide with antibacterial properties and protective film formation that is used as an edible coating (Peng and Li 2014). In the study by Yehia et al. (2021), natural antimicrobial compounds were used to increase the shelf life of fresh camel meat and control *Campylobacter jejuni* contamination. In this study, Citrox, which includes a range of bioflavonoids extracted from citrus fruits and can degrade bacterial biofilms and destroy cell walls, along with chitosan, which is a positively charged amino‐polysaccharide that binds to negatively charged molecules and has antimicrobial activity against gram‐positive and gram‐negative bacteria, was studied in camel meat samples during storage for 30 days under vacuum conditions (VP) at temperatures of 4°C and 10°C. During the storage period, total microbial count (TVC), total volatile base nitrogen (TVBN), and pH were measured every 3 days. The results showed that the combination of Citrox (1% and 2%) with 1% chitosan was more effective than Citrox alone in reducing the growth of *C. jejuni*, and produced an approximate 4.0 logarithmic reduction at 4°C and 3.5 logarithmic reduction at 10°C. Also, the combined treatment, in addition to microbial inhibition, improved the physicochemical quality of camel meat and allowed for an increase in shelf life of more than 30 days at 4°C (Yehia et al. 2021).

The use of Arabic Gum as a natural and safe hydrocolloid has been able to significantly increase the shelf life of camel meat. Studies have shown that 10% Arabic gum dispersion has a stronger antibacterial effect than some antibiotics, especially against *Klebsiella pneumoniae* , and has been able to increase the microbial and sensory shelf life of camel meat from 1 day to 14 days, while concentrations of 5% and 2.5% increased the shelf life to 11 and 8 days, respectively (Elsharawy et al. 2026).

Nanocomposite films of nanomontmorillonite‐chitosan (MMT‐Ch) and nanomontmorillonite‐carboxymethylcellulose (MMT‐CMC), incorporating 0.5%–2% *Ziziphora clinopodioides* essential oil, alone or with 1% *Ficus carica* extract, show promise as active packaging for ground camel meat. These films significantly inhibited *Listeria monocytogenes* and *Escherichia coli* O157:H7 growth during cold storage. Specifically, CMC‐MMT + 2% ZEO + 1% FCH and Ch‐MMT + 2% ZEO + 1% FCH films reduced microbial populations by 1–4 log CFU/g compared to controls. These treatments also slowed increases in TVB‐N, pH, peroxide value, protein carbonyls, and TBARS, while improving odor, color, and overall acceptability. Thus, these nanocomposite films could effectively extend the shelf life of camel meat (Khezrian and Shahbazi 2018). Among the valuable studies on the application of biodegradable films in improving the oxidative stability of camel meat, basil seed gum‐based films containing antioxidant compounds, especially in the form of nanoemulsion, are considered a novel approach to increase the shelf life of meat products. In this study, the combined effects of resveratrol and clove essential oil in basil seed gum‐based films were investigated. The results showed that nanoemulsion films had higher antioxidant activity than conventional films, and the synergistic interaction between resveratrol and clove essential oil enhanced this property. The use of nanoemulsion film containing resveratrol (4 μg/mL) + clove (10 mg/mL) in the packaging of ground camel meat during 20 days of storage at 4°C improved oxidative stability indices; so that the total carbonyl values (−0.84 nmol/mg protein), peroxide (4.03 meq/kg lipid) and (1.03 mg MDA/kg TBARS) decreased compared to the control group. Also, the sensory evaluation results showed a higher overall acceptance in the treated samples. Overall, the findings indicate that bioactive films based on basil seed gum containing resveratrol and clove essential oil can be used as a new method in the packaging of meat and meat products to control oxidation and improve sensory quality (Ansarian et al. 2022).

Beyond physical packaging, the direct incorporation of preservatives into the meat matrix offers an additional layer of protection. These additives, whether natural or synthetic, target microbial growth and oxidation at a chemical level. The subsequent section categorizes and evaluates these preservative agents.

## 8 Use of Additives and Preservatives

### 8.1 Natural Preservatives

#### 8.1.1 Herbs and Essential Oils

Essential oils and plant extracts are known for their remarkable antimicrobial properties. These essential oils and extracts, which are obtained from various plants, contain natural compounds that can inhibit or kill the growth of microorganisms such as bacteria, viruses, and fungi (Preedy 2015). Numerous scientific studies have investigated the antimicrobial effects of essential oils (Table 3). They have shown efficacy against a wide range of pathogens, including common bacteria such as *Escherichia coli* , *Staphylococcus aureus* , and *Salmonella*, as well as viruses such as influenza and herpes (Tajkarimi et al. 2010). The antimicrobial activity of essential oils and plant extracts is attributed to their bioactive compounds such as terpenes, phenols, and aldehydes. These compounds can disrupt microbial cell membranes, inhibit enzyme activity, and interfere with essential metabolic processes, leading to the suppression of microbial growth. In addition to antimicrobial properties, essential oils have also shown antifungal effects (Preedy 2015). Accordingly, they have shown potential as natural food preservatives due to their antimicrobial properties. They can help inhibit the growth of spoilage microorganisms, extend the shelf life of food products, and reduce the risk of foodborne illnesses (Murbach Teles Andrade et al. 2014). Essential oils can be used directly or in combination with other preservation techniques, such as packaging and refrigeration, to increase effectiveness. They can be applied to foods through methods such as spraying, dipping, or incorporating into edible coatings and films (Osaili et al. 2021).

**TABLE 3:** Application of preservatives in camel meat products.

Type of preservative	Treatment	Findings	References
Peppermint essential oil ( *Mentha spicata* )	0.5%–1.5% in ground camel meat, 12 days at 4°C	Reduced Pseudomonas spp., Enterobacteriaceae, *L. monocytogenes* load by 1–4 log CFU/g, improved odor and color, reduced oxidative spoilage	Shahbazi et al. (2018)
Plant essential oils (carvacrol, cinnamaldehyde, thymol)	1%–2% in marinated meat, at 4°C and 10°C	Delayed spoilage microorganisms; stronger effect at 10°C	Osaili et al. (2021)
Marjoram, rosemary, sage powder	3% in ground meat, 12 days at 5°C	Reduced TBARS and microbial counts, improved sensory quality	Kamel (2013)
Fresh garlic	5%–25% (w/w) in camel meat, stored at 20°C–22°C, 12°C and 2°C–3°C	Shelf life increased 2–4 times, complete inhibition at higher doses	Al‐Delaimy and Barakat (1971)
Citric acid	0.5%–1.5% in fresh slices, 15 days at 5°C	Lowered pH and oxidation, shelf life extended up to 15 days	Abd Elgadir et al. (2025)
Chitosan + rosemary essential oil + sodium lactate	1% Chitosan, 0.2% rosemary essential oil, 2% sodium lactate in ground meat	Improved physicochemical and sensory quality, reduced microbial load	Hussein et al. (2021)
Cumin essential oil ( *Cuminum cyminum* L.)	0.2% in camel sausage, 15 days at 4°C	Extended shelf life up to 15 days, reduced microbial growth	Moghimi et al. (2021)
Gingerol (1.5%) + Nisin (2.5%)	Fresh camel meat	Reduced microbial load ~50%, improved quality	Tang et al. (2020)
Tannic acid or catechin (200 mg/kg)	Camel meat, 9 days refrigerated storage	Reduced mesophilic and psychrophilic bacterial counts by 1 log unit; extended shelf life up to 9 days	Maqsood, Abushelaibi, Manheem, Al Rashedi, and Kadim (2015)
Lactic acid 2%	Camel meat, immersion in solution, stored at 4°C	6	Atika et al. (2014)
Fresh leek ( *Allium ampeloprasum* ) as natural nitrite source	Added at different levels in camel sausages compared to nitrite‐added control	Increased nitrite content, no effect on cooking loss; significantly reduced TBA after 45 days; prolonged frozen storage stability	Engy and Mohamed (2018)
Potassium sorbate	0.3% in ground camel meat, stored at 4°C ± 1°C	Significantly reduced microbial load, especially molds and yeasts; shelf life extended up to 8 days; maintained pH and surface color	Hussein et al. (2012)
Combined biopreservation (Nisin + *Olea europaea* subsp. *laperrinei* extract)	Camel steaks under MAP (80% O_2_ + 20% CO_2_), stored 30 days at 1°C ± 1°C	Strong inhibition of spoilage bacteria (psychrotrophs esp. *Pseudomonas* spp.), reduced lipid oxidation, preserved redness, extended shelf life from ~7 to > 20 days Lower TBARS and MetMb compared to control; no negative effect on tenderness; higher sensory scores	Djenane et al. (2020)
Chitosan film + nanoemulsion of black cumin EO + lemon balm extract	2% chitosan, 2.5%–5% black cumin EO, 4% lemon balm extract, stored 16 days at 4°C	Strong antioxidant and antimicrobial effect against *L. monocytogenes* ; reduced TBARS, pH and bacterial counts; improved film antioxidant properties	Ebrahimian and Mohsenzadeh (2023)
Herbal additives (thyme, rosemary, clove, ginger)	3% of each in semi‐dried processed camel meat, dried at 35°C ± 3°C for 36 h	Improved microbial and sensory quality; thyme and rosemary most effective; proposed as natural preservatives	Aljabeili et al. (2016)
Spirulina (*Arthrospira platensis*) powder	100–500 mg/kg in camel merguez sausages, vacuum‐packed, 35 days at 1°C ± 1°C	Extended shelf life from 5 (control) to 20–35 days; reduced TBARS, TVB‐N, psychrotrophs; improved color and sensory quality; stimulated probiotics and inhibited pathogens ( *S. aureus* , *E. coli* O157:H7)	Djenane et al. (2024), Kalalou et al. (2004)
Ultrasound‐assisted hydrolysis of camel skin gelatin	Applied as antioxidant/antimicrobial hydrolysates, used as edible coatings on chilled camel meat	Rich in antioxidant amino acids; inhibited lipid oxidation, reduced TVB‐N, microbial growth, stabilized pH; improved shelf life	Hamrouni et al. (2023)
Stinging nettle ( *Urtica dioica* L.) essential oil (free and nanoliposome form)	Applied to ground camel meat, chilled storage	Nanoliposomes improved EO stability and antimicrobial activity; inhibited *L. monocytogenes* and *E. coli* ; reduced spoilage indices (pH, TBARS, TVB‐N); improved sensory quality	Shabani et al. (2023)

Shahbazi et al. (2018) added spearmint essential oil ( *Mentha spicata* ; MSO) to raw ground camel meat to increase its shelf life and evaluate its antimicrobial activity against *Listeria monocytogenes* during 12 days of refrigerated storage. The final microbial population (mesophilic and psychrotrophic bacteria, *Pseudomonas* spp., Enterobacteriaceae, and *L. monocytogenes* ) in the treated samples was about 1–4 log CFU/g lower than the control group. The initial total volatile free nitrogen content reached 39.2 mg/100 g after 12 days in the control group, while it remained less than 25 mg/100 g in the samples treated with 1% and 1.5% MSO. Also, at the end of the storage period, the peroxide value in the control samples was higher than in the treated samples. In addition, during 12 days of storage, the odor, color, and appearance of ground camel meat treated with 0.5% MSO were superior (Shahbazi et al. 2018).

Osaili et al. (2021) investigated the antimicrobial effect of plant essential oils including carvacrol, cinnamaldehyde, and thymol at concentrations of 1% and 2% on the growth of spoilage microorganisms in marinated camel meat during storage at temperatures of 4°C and 10°C. However, the essential oils showed a greater inhibitory effect at higher temperatures (10°C). At 10°C, the greatest reduction in the total count of mesophilic bacteria, yeast and mold, mesophilic lactic acid bacteria, Enterobacteriaceae, and Pseudomonas was 1.2, 1.4, 2.1, 3.1, and 4.8 log CFU/g, respectively. The use of 2% essential oil in marinated camel meat and its storage under aerobic conditions and higher than the permissible temperature was able to significantly delay spoilage (Osaili et al. 2021).

One of the prominent studies on the preservation effect of plant essential oils in camel meat was conducted by Kamel (2013). In this study, the improvement of the quality of ground camel meat was investigated by adding 3% (w/w) of marjoram, rosemary and sage powder during storage at refrigerated temperature. Ground camel meat was stored at 5°C and periodically examined for indicators of lipid oxidation (TBARS), total aerobic bacteria count, coliform bacteria, psychrotrophic bacteria, yeast and mold, as well as sensory characteristics during 12 days of storage. All tested plants significantly reduced TBARS values after 5 and 12 days compared to the control sample and improved sensory properties. Marjoram reduced the total aerobic bacteria count by one logarithm compared to rosemary and sage. Also, yeast and mold counts in samples treated with marjoram were lower than other samples during the storage period. Overall, marjoram showed the highest antimicrobial activity compared to other plants and can be considered as a promising option in food safety and preservation (Kamel 2013). In addition, Hussein et al. (2021) showed that the addition of chitosan (1%), rosemary essential oil (0.2%), and sodium lactate (2%) could improve the organoleptic, chemical, and microbial quality of ground camel meat during refrigerated storage (Hussein et al. 2021). Among them, sodium lactate had the greatest effect in reducing the microbial load (Psychrotrophs, Enterobacteriaceae, and Pseudomonas) and also inhibiting the formation of biogenic amines. While synthetic antimicrobial preservatives can be effective, the demand for natural preservatives is increasing. Herbs such as thyme, rosemary, and cinnamon were observed to significantly increase the shelf life of meat, with thyme having the greatest effect, extending the shelf life by up to 60 days. It was also found that herbal compounds had a greater effect on the storage stability of meat compared to single herbs (Asif et al. 2024).

Additionally, fresh garlic has been used as an antimicrobial and preservative agent to increase the shelf life of fresh camel meat. In this study, three storage temperatures were used, including ambient temperature (20°C–22°C), incubator (12°C), and refrigerator (−2°C–3°C). Regardless of the storage temperature, treatment of lean camel meat with 5%, 10%, and 15% (wt.) of freshly minced garlic increased the shelf life by two, three, and more than four times, respectively, compared to the control samples. After 4 days of storage at ambient temperature, 12 days in the incubator, and 28 days in the refrigerator, it was found that treatment with 15% and 25% garlic resulted in complete inhibition of microbial growth and no signs of organoleptic spoilage were observed in the meat. After frying for 15 min, the treated meat samples were considered acceptable in terms of taste by local people in Saudi Arabia (Al‐Delaimy and Barakat 1971). Jumaa conducted a study to evaluate the qualitative and chemical properties of processed meat products (camel meat burgers) stored at 4°C for 12 days. These products were treated with three different concentrations of peppermint oil extract. Chemical and qualitative tests were performed in refrigerated storage. The results clearly showed that the treatment containing 1% peppermint oil had a significant advantage over the other treatments in maintaining the chemical and qualitative properties of the samples within acceptable limits after 12 days of storage at 4°C (Jumaa, n.d.).

#### 8.1.2 Organic Acids

Edible organic acids have the potential to increase the shelf life of meat because they improve the overall quality of meat by controlling harmful bacteria and preventing oxidative spoilage. Edible organic acids and their salts are commonly used as food additives because they are safe for human consumption and are generally recognized as safe (GRAS) and can be safely used in food (Ben Braïek and Smaoui 2021). Some studies have shown that the use of citric acid can act as a simple and effective method to increase the shelf life of camel meat. Fresh camel meat slices were treated with different concentrations of citric acid (0.5%, 1%, and 1.5%) and then stored under refrigerated conditions (5°C) for 15 days. Treatment with citric acid caused a decrease in pH (from 5.3 in the control sample to the range of 4.2–4.47) and a decrease in water activity. Also, the TBARS, which indicates lipid oxidation, was significantly lower in the treated samples than in the control samples; especially at a concentration of 1% citric acid, the lowest value was recorded. Microbiologically, the total microbial count in the treated samples, especially at higher concentrations, decreased significantly and remained below the critical level (10^7^ CFU/g) during storage. Based on these results, treatment with citric acid up to a concentration of 1.5% was able to significantly increase the microbiological shelf life and sensory quality of fresh camel meat for up to 15 days (Abd Elgadir et al. 2025). Also, according to Teshome et al. (2022), the effectiveness of natural antimicrobial compounds such as edible organic acids in meat coating applications is influenced by factors such as food composition, processing methods and storage conditions. However, they reported that several strategies should be implemented to improve the applicability of natural preservatives, including the combination of different preservatives and food preservation methods such as active packaging systems and encapsulation (Teshome et al. 2022). In this regard, Bhagath and Manjula (2019) reported that the application of organic acids in edible meat coatings can reduce the microbial count of fresh meat. Similar findings were previously observed by Siragusa and Dickson (1992); they stated that the addition of edible organic acids such as lactic acid (1.7%) and acetic acid (2%) to edible coatings can reduce the microbial count by 1.5 and 0.25 log, respectively (Siragusa and Dickson 1992). However, in a study conducted by Maqsood, Abushelaibi, Manheem, Al Rashedi, and Kadim (2015), the addition of 200 mg/kg tannic acid or catechin to camel meat caused a one‐log reduction in the total mesophilic and psychrophile bacterial counts after 9 days of refrigerated storage. As a result, the shelf life of the meat was increased by up to 9 days (Maqsood, Abushelaibi, Manheem, Al Rashedi, and Kadim 2015).

Many other researches have been conducted to develop different storage methods to increase the shelf life of fresh camel meat. Atika et al. (2014) investigated the combined effect of 2% lactic acid solution and refrigerator temperature on the shelf life of fresh camel meat. The samples were immersed in the acid solution and stored at 4°C. They found that the shelf life of the meat could be increased by up to 9 days (Atika et al. 2014). In another study, it was found that storing fresh camel meat at 4°C for 12 days could increase the shelf life of the product without negatively affecting its sensory acceptance. Moghimi et al. (2021) used 0.2% cumin ( *Cuminum cyminum* L.) essential oil as a natural preservative and applied it to fresh camel sausage for 15 days at 4°C. The results showed that cumin essential oil can significantly increase the shelf life of fresh camel sausage by up to 15 days (Moghimi et al. 2021). Tang et al. (2020) found that treating fresh camel meat with gingerol (1.5%) and nisin (2.5%) can reduce the total microbial count by 58.35% and 47.76%, respectively, while improving the quality of the meat (Tang et al. 2020).

### 8.2 Synthetic Preservatives

Nitrites and nitrates are important in the curing of meat products, in giving the meat its characteristic color. In addition, nitrite acts as a bacteriostatic and bactericidal agent in cured meats (Fista et al. 2004). Nitrites are highly inhibitory to anaerobic bacteria, especially *Clostridium botulinum* , and help control other microorganisms, such as *Listeria monocytogenes*. They are also a potent antioxidant. Over the past 20 years, nitrite has been linked to methemoglobinemia and the formation of carcinogenic nitrosamines in humans. This has led to restrictions on the amount of nitrate and nitrite in foods (Fista et al. 2004). Fresh chives are a good source of nitrates, flavonoids, polysaccharides, and glucosinolates, as well as several allosulfur compounds. Engy and Mohamed (2018) investigated the effect of adding different amounts of chives as a natural source of nitrite in the preparation of camel sausage and studied its effect on the quality characteristics of the prepared product compared to camel sausage containing nitrite. With increasing amounts of chives, the nitrite content increased. The level of chives did not affect the cooking loss of sausage samples. TBA values in the formulation samples decreased significantly after 45 days, and this continued with the lengthening of the frozen storage period (Engy and Mohamed 2018).

Another chemical preservative in camel meat is potassium sorbate. Some studies have shown that the use of potassium sorbate in low amounts can effectively increase the shelf life of camel meat (Engy and Mohamed 2018). In one study, the addition of 0.3% potassium sorbate to ground camel meat significantly reduced the microbial load, especially in the counts of molds and yeasts. Following this treatment, the microbiological shelf life of meat under refrigerated conditions increased to about 8 days, while in the control samples this period was shorter. The treatment also maintained the pH level and reduced surface discoloration. From a practical point of view, this method is simple, cheap and available and can be recommended as a suitable method for improving the microbial quality and safety of camel meat (Hussein et al. 2012).

While conventional additives are effective, consumer demand for minimally processed foods has driven the development of advanced technologies. These emerging methods utilize physical or biological interventions to achieve safety without relying heavily on chemical additives. The following section highlights these cutting‐edge innovations.

## 9 Advanced Technologies

### 9.1 Nanotechnology

**FIGURE 4:**
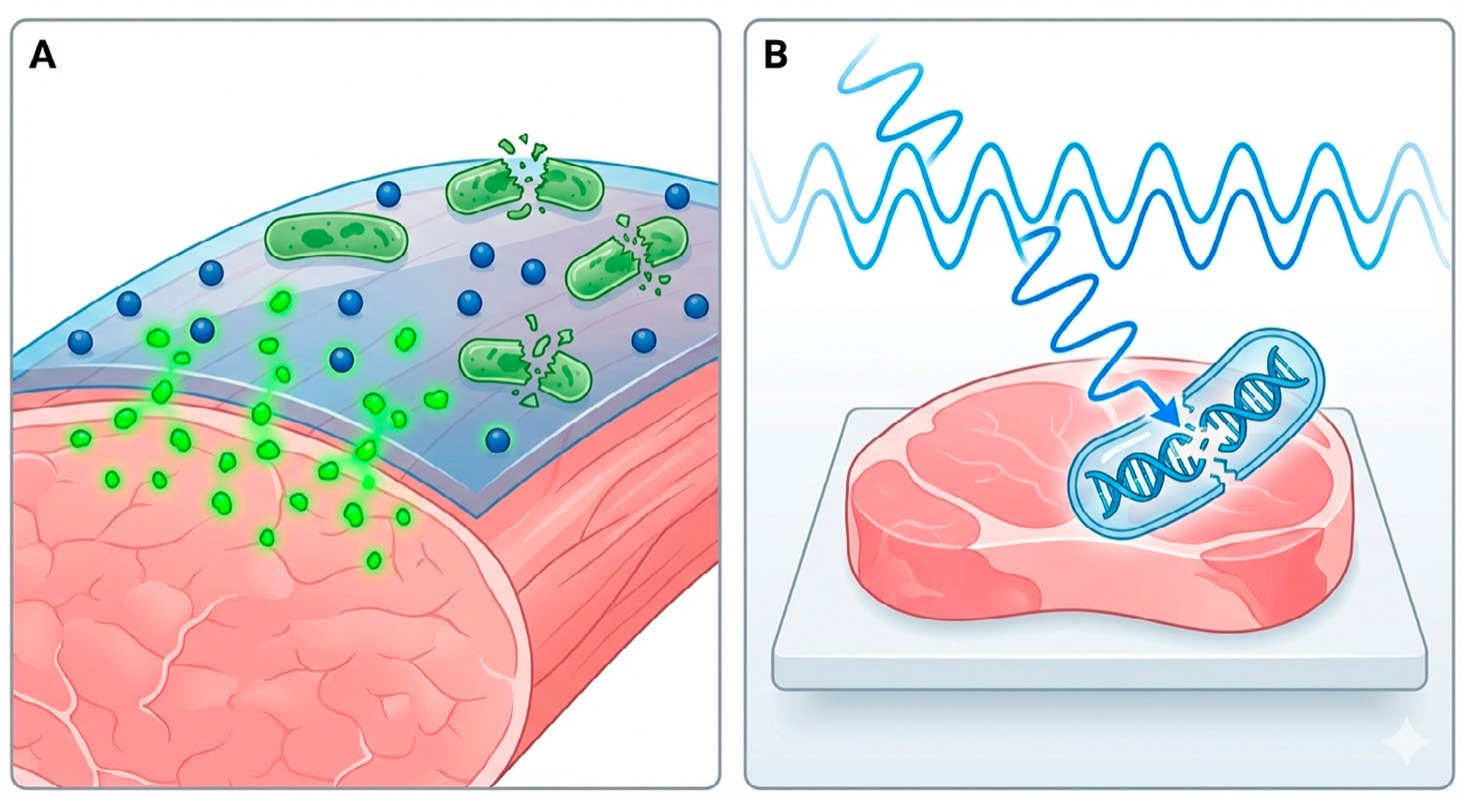
Advanced technologies in camel meat preservation: (A) nanotechnology; (B) irradiation.

The use of silver nanoparticles, nano‐chitosan and nanomontmorillonite in packaging films can provide antibacterial and antioxidant properties (Figure 4). Recent studies have shown that the use of nanocomposite films based on nanomontmorillonite‐chitosan (MMT‐Ch) and nanomontmorillonite‐carboxymethylcellulose (MMT‐CMC) containing *Ziziphora clinopodioides* essential oil at concentrations of 0.5%, 1% and 2%, alone or in combination with 1% *Ficus carica* extract, can be used as an active packaging system for ground camel meat. These films were able to significantly reduce the growth of *Listeria monocytogenes* and *Escherichia coli* O157:H7 during cold storage. These packaging films caused a reduction in microbial population by about 1 to 4 log CFU/g compared to the control group. In addition, these treatments slowed down the increase in TVB‐N, pH, peroxide value, protein carbonyl and TBARS and improved sensory properties including odor, color and overall acceptability. Accordingly, such nanocomposite films can be introduced as promising packaging materials to increase the shelf life of camel meat (Khezrian and Shahbazi 2018). In addition, other studies have shown that the use of plant essential oils, especially in the form of nanostructured systems, can be an effective solution to increase the shelf life of red meats (Shabani et al. 2023). In this context, nettle essential oil ( *Urtica dioica* L.) was investigated in two forms, free and nanoliposomal, in ground camel meat. The results showed that nanoliposomes were able to improve the physicochemical properties of the essential oil, including particle size, stability, and encapsulation efficiency, and enhanced its antimicrobial activities against *Listeria monocytogenes* and *Escherichia coli* . Also, treatment of camel meat with nanoliposomal essential oil reduced spoilage indices including pH, total volatile base nitrogen (TVB‐N), and lipid oxidation, along with improving sensory properties during cold storage. These findings indicate that nettle essential oil, especially in nanoliposomal form, can be used as a natural preservative and an alternative to chemical additives in the packaging and storage of camel meat.

### 9.2 Irradiation

Gamma irradiation can improve the microbial quality of camel meat (Figure 4). The total microbial load decreased with increasing irradiation dose (2–6 kGy), and this effect was greater during storage at 4°C (Al‐Bachir and Zeinou 2009). The coliform count decreased from 3.15 to < 1 at a dose ≥ 2 kGy, and the coliform count remained constant at this level during 6 days of storage (Al‐Bachir and Zeinou 2009). Irradiation at doses of 1.5 and 3.0 kGy increased the microbial life of fresh camel meat to 6 and 12 days, respectively, and had no effect on proximate composition, total volatile nitrogen (TVN), cooking weight loss, and sensory characteristics (Fallah et al. 2010). These findings confirm a previous report that irradiation in the dose range of 2–6 kGy had no effect on the sensory properties of camel meat (Al‐Bachir and Zeinou 2009). The acceptance period of meat after irradiation with doses of 1.5 and 3.0 kGy increased from 7 days to 15 and 21 days, respectively (Fallah et al. 2010). Although Al‐Bachir and Zeinou found that irradiation intensity in the range of 2–6 kGy had no effect on fat oxidation of camel meat (Al‐Bachir and Zeinou 2009); Fallah et al. (2010) reported that fat oxidation increased almost twofold at a dose of 3.0 kGy. Based on the above information, irradiation is an effective preservation method, combined with hygienic production principles, to extend the shelf life of fresh camel meat (Fallah et al. 2010).

## 10 Biological Methods

In an effort to reconcile consumer demands with safety standards, the combination of modern technologies including biological microbicidal systems such as lactic acid bacteria (LAB) has replaced traditional methods of controlling microbial hazards in foods. The use of LAB is not new and has been used for centuries for food preservation. In this context, the preservation of fermented and cooked meat with LAB has been common for many years (Kalalou et al. 2004). Various strains have been reported to exhibit antagonistic behavior against pathogenic and spoilage microorganisms in these products. These antagonistic properties are usually achieved through the production of one or more antimicrobial metabolites such as organic acids (lactic and acetic), hydrogen peroxide, antimicrobial peptides (bacteriocins), and bioactive enzymes (Kalalou et al. 2004; Millette et al. 2007). Kalalou et al. (2010) have shown in their studies that the use of lactic acid bacteria, especially *Lactobacillus plantarum* species, can act as an effective biological method to reduce the microbial load and increase the safety of fresh camel meat. In this study, fresh camel meat was inoculated with *E. coli* O157:H7 and then treated with *L. plantarum* and stored at 10°C for 4 days. The results showed that treatment with this bacterium significantly reduced the survival of *E. coli* O157:H7 by about 2 log CFU/g and at the end of the storage period under vacuum conditions, the number of harmful bacteria in the treated samples was lower than that in the control samples. Also, the count of lactic acid bacteria increased during the storage period and the lactic acid flora developed spontaneously. These findings indicate that the use of probiotic bacteria such as *L. plantarum* can be recommended as a natural and safe strategy to improve the microbial quality and enhance the safety of fresh camel meat (Kalalou et al. 2010). A study by Wambui et al. (2017) on raw camel meat showed that this meat may carry pathogenic bacteria of the Enterobacteriaceae family, which are of great importance in food safety. In this study, isolates of lactic acid bacteria adapted to the camel meat production environment were isolated from the traditional fermented beverage Susak and their ability to inhibit the growth of some members of the Enterobacteriaceae was investigated. The results showed that LAB were able to inhibit *Citrobacter* spp., *Shigella* spp. and some *E. coli* isolates, while *Salmonella* spp. were not affected. The mean diameter of the inhibition zone was reported to be between 8.5 and 12.5 mm, and no significant difference was observed between the inhibited organisms. These findings suggest that LAB can be used as a potential protective culture in improving the safety and quality of raw camel meat and has the potential to reduce the microbial risks associated with the consumption of this product (Wambui et al. 2017).

## 11 Combined Methods

Djenane et al. (2020) investigated the effect of combining two natural preservatives, nisin (a bacteriocin with antibacterial properties) and Algerian wild olive leaf extract ( *Olea europaea* subsp. laperrinei), on increasing the shelf life of camel meat packaged under Modified atmosphere packaging conditions. Fresh camel meat was packaged with a gas composition of 80% O_2_ and 20% CO_2_. The combination of nisin + olive leaf extract had the greatest inhibitory effect on the growth of spoilage bacteria such as *Pseudomonas* spp. It also significantly reduced fat oxidation and maintained the red color of the meat during storage. In addition, the shelf life of the meat increased from about 7 days (control) to more than 20 days (Djenane et al. 2020).

The effect of combined biopreservation treatment with *Olea europaea* subspecies laperrinei leaf extract (laper.OLE) and nisin on the quality characteristics of camel steaks packaged in modified atmosphere conditions with high concentrations of O_2_ (80%) and CO_2_ (20%) during long‐term storage at refrigerated temperature (1°C ± 1°C) was also investigated. Based on high‐performance liquid chromatography and diode array detector (HPLC/DAD) analysis, the dominant phenolic compound in the chemical structure of laper.OLE was oleopene (63.03%). Camel steaks treated with laper.OLE had lower concentrations of thiobarbituric acid‐reactive compounds (TBA‐RSs) during 30 days of storage. The level of surface metmyoglobin (MetMb) in laper.OLE‐treated samples increased at a lower rate than in control samples. None of the modified atmosphere packaging methods or biopreservation treatments significantly altered meat tenderness (based on Warner‐Brassler shear force, WBSF) compared to the control samples. After 30 days of storage, the number of psychrotrophic bacteria and *Pseudomonas* spp. in camel steaks treated with the combination of laper.OLE and nisin was significantly lower than in untreated samples. In addition, samples treated with laper.OLE received a higher score in bitterness acceptance. Overall, the use of combined biopreservation methods can be a sustainable solution for preserving and improving the quality characteristics of camel meat in arid regions (Djenane et al. 2020).

Among other combined studies in this field, a study was conducted to investigate the antimicrobial and antioxidant effects of chitosan film containing nanoemulsion of *Melissa officinalis* extract and black cumin essential oil against *Listeria monocytogenes* bacteria inoculated into camel meat. The studied films were prepared using 2% chitosan and 2.5% and 5% concentrations of black cumin essential oil and 4% Melissa extract nanoemulsion. The antioxidant and antimicrobial effects of coated camel meat pieces were evaluated during 16 days of storage at 4°C with a 4‐day interval (0, 4, 8, 12, 16). The coated pieces were chemically evaluated. The most abundant compounds of black cumin included cumin aldehyde (37.24%), gamma terpinene (99.19%) and para‐cymene (719.9%). The minimum inhibitory concentration of Melissa extract and black cumin against *Listeria monocytogenes* was determined to be 1% and 0.25%, respectively. The results of evaluating the antioxidant properties of the films by the DPPH method showed that the addition of essential oil and extract increased the antioxidant properties of the films. In the study of the antimicrobial effect of films by disk diffusion method, the largest diameter of the growth inhibition zone (17.15 ± 0.16) was related to the chitosan film containing 5% black cumin essential oil nanoemulsion and 4% Melissa extract. The average bacterial count in the control treatment during the 16‐day period was higher than the other treatments studied. TBARS results indicated greater antioxidant properties in the films containing black cumin essential oil and Melissa extract compared to the control sample. The pH level in the samples coated with chitosan film containing 5% black cumin essential oil nanoemulsion and 4% Melissa extract was lower than the other samples. Overall, the prepared films had good antimicrobial and antioxidant properties against *Listeria monocytogenes* , which increased with the addition of plant compounds (Ebrahimian and Mohsenzadeh 2023).

Among the studies that used combined treatments for the preservation of camel meat, the study by Aljabeili et al. (2016) aimed to determine the quality characteristics of cured and semi‐dried camel meat containing some medicinal plants including thyme, rosemary, cloves, and ginger as natural preservatives. Camel meat samples were cut and divided into five groups; one group was considered a control sample that was treated only with the curing mixture (including black pepper, cumin, spice mixture, dried onion, dried garlic, and salt). To investigate the effect of different sources of natural preservatives including thyme, rosemary, cloves, and ginger, 3% of each of these preservatives was combined with the above curing mixture and used to cure four groups of camel meat cuts. After the curing process, camel meat slices (control and treated with natural preservatives) were subjected to drying process at 35°C ± 3°C for 36 h in a drying cabinet. The quality characteristics of the prepared semi‐dried camel meat including chemical composition, microbiological characteristics, and sensory characteristics were evaluated. Based on the results of microbiological and sensory characteristics, it can be suggested that the selected medicinal plants, especially thyme and rosemary, can be used as natural preservatives in the preparation of semi‐dried camel meat without causing negative effects (Aljabeili et al. 2016).

Other studies in the field of using combined preservation methods in camel meat products include the study by Djenane et al. (2024). This study has shown that the addition of *Spirulina* powder (SP) to merguez type sausage made from camel meat can improve the physicochemical, microbial and sensory properties of the product and increase its prebiotic potential. In this study, different sausage formulations were prepared with 0, 100, 250, and 500 mg SP/kg and stored under vacuum packaging at 1°C ± 1°C for 35 days. The results showed that the addition of SP increased the shelf life of the sausage from 20 to 35 days, while the control group spoiled by the fifth day. The positive effect of adding SP became more prominent with increasing its concentration and the TBARS, CIE a*, TVB‐N indices, psychrotrophic bacteria count and overall acceptance score were improved. In addition, SP stimulated the growth of probiotic strains and these strains were able to inhibit the growth of pathogenic bacteria such as *S. aureus* and *E. coli* O157:H7 (Djenane et al. 2024). Therefore, the use of SP as a natural and healthy material (Clean Label) can be a source of beneficial compounds for human health and provide a new solution for the production of camel sausage with better acceptance (Kalalou et al. 2004).

Recent studies have shown that the use of ultrasound pretreatment to enhance the enzymatic hydrolysis of camel skin gelatin can significantly increase the antioxidant and antimicrobial properties of this compound. The hydrolysate obtained from this process contains antioxidant amino acids such as methionine, cysteine, histidine, and phenylalanine and has shown high ability in free radical scavenging, reducing activity, and preventing lipid oxidation. Also, the use of gelatin coatings containing these products on chilled camel meat has reduced weight loss, stabilized pH, limited lipid oxidation, reduced total basic nitrogen (TVB‐N), and inhibited microbial growth. Overall, these results indicate that camel skin gelatin after ultrasound treatment and enzymatic hydrolysis can be used as a natural alternative to synthetic additives in preserving and increasing the shelf life of meat products (Hamrouni et al. 2023).

Having reviewed the spectrum of preservation strategies ranging from traditional salting to advanced nanotechnology, it is evident that a multi‐hurdle approach is most effective. The following section synthesizes these findings to offer conclusions and outlines future directions for the camel meat industry.

## 12 Processed Products

Fresh sausage is one of the oldest meat products in the world, made from beef, buffalo, sheep, lamb, goat, camel, poultry, fish, and horse. Due to its high water activity, high pH, lack of chemical preservatives, and no heat treatment, it is susceptible to lipid oxidation and microbial spoilage (Kalalou et al. 2004). The use of plant essential oils is a new approach to the biopreservation of fresh sausage. Essential oils, also known as volatile oils or ethereals, are complex, aromatic oil mixtures of volatile compounds that are usually extracted from plants by steam distillation. These compounds are classified as natural preservatives and have the potential to inhibit the growth of spoilage and pathogenic bacteria (Carballo et al. 2019). Fresh sausage has a short shelf life and is easily spoiled in cold storage conditions. A study by Moghimi et al. (2021) aimed to investigate the effect of *Cuminum cyminum* L. essential oil on increasing the shelf life of fresh camel sausage during storage at 4°C for 15 days. Based on microbial findings, the addition of CCEO significantly delayed microbial growth in sausage compared to the control group. The mesophilic bacteria count in the control samples reached the upper limit of the microbial limit (7 log CFU/g) on the fifth day, while this value was observed in the samples containing 0.05% and 0.1% CCEO on the seventh day and in the samples containing 0.2% CCEO on the fifteenth day. Based on the chemical findings, the total volatile base nitrogen (TVB‐N) content in the control samples increased to 39.75 mg/100 g on the last day of storage. At the end of the study, a significant decrease (about 6.29–11.85 mg/100 g) in the final TVB‐N content was observed in the samples treated with CCEO compared to the control (*p* < 0.05). Also, the peroxide value (PV) in the control samples was 4.49 mEq/1000 g fat, while this value remained lower in the treated samples at the end of the study (3.25 mEq/1000 g fat). In terms of sensory properties, the addition of 0.05% and 0.1% CCEO had slight negative effects on the sensory properties of the samples (*p* > 0.05). Based on the results, the use of CCEO in fresh camel sausage is a practical method to increase the shelf life of this product (Moghimi et al. 2021).

In addition, studies have shown that the use of lactic acid bacteria can be used as an effective biological method to increase the shelf life of fermented camel sausage in hot regions (Kalalou et al. 2004). In one study, fresh camel meat was inoculated with *Lactobacillus plantarum* strain after deboning and fat separation, and stuffed into natural casings in the form of sausages and dried at 15°C–18°C with a relative humidity of 70%–80%. The results showed that the counts of coliforms, enterococci and yeasts were completely eliminated after 21 days and the total microbial count decreased significantly during the ripening period. Proteolytic and lipolytic microorganisms were also reduced to less than 1 cfu/g and 3 × 10^4^ cfu/g, respectively. The pH of the meat reached 4.5 and the water activity reached 0.7. These findings indicate that the use of LAB as a bioprotection agent can improve the microbial and chemical quality of camel sausage and help increase the safety and shelf life of the product (Kalalou et al. 2004). Al‐Juhaimi et al. (2018) studied the use of Argel leaf powder in improving the quality of camel meat products and found that Argel leaf powder (ALP) has been considered as a natural additive in meat products due to its valuable phytochemical compounds and high antioxidant activity. This material contains a significant amount of total phenolic compounds (about 1262.5 mg GAE/100 g) and anthocyanins (60.11 μmol/g) and exhibits high DPPH radical scavenging activity (about 86.85%). The main phenolic compounds identified in ALP include isorhamnetin, catechin, catechol, gallic acid, and protocatechuic acid, each of which plays an important role in its antioxidant and antimicrobial properties. The addition of ALP to camel burger formulation improved various product characteristics. With increasing ALP concentration, protein and fat content, cooking efficiency, moisture and fat retention, total phenolic content, antioxidant activity (DPPH), redness (a), yellowness (b), and microbial stability increased. In contrast, the lipid oxidation indices (TBARS), lightness (L), and shrinkage decreased. During the storage period, camel burgers containing ALP showed greater stability in physicochemical, oxidative, microbial and sensory properties. Overall, these results indicate that Argel leaf powder at a concentration of 4 to 6% can be used as a natural additive, antioxidant and antimicrobial effective in maintaining the quality and increasing the shelf life of camel meat products (Al‐Juhaimi et al. 2018).

## 13 Conclusion

Camel meat, as a valuable source of animal protein, is not only of particular importance in nutrition and food security in arid and semi‐arid regions, but can also gain a significant position in global markets. However, microbial spoilage and physicochemical changes are the most important challenges limiting its shelf life. A review of previous studies showed that various methods have been used to increase the shelf life of camel meat; from traditional methods such as salting, drying, and smoking, to modern technologies such as thermal methods, vacuum packaging, and modified atmosphere. Also, the use of natural (such as plant extracts and essential oils) and synthetic additives and emerging technologies such as irradiation, nanotechnology, and ultrasound have each played a role in controlling spoilage and improving the quality of this product. Despite these advances, scientific evidence shows that many new technologies have not yet been widely investigated in camel meat. In particular, methods such as super‐chilling and high hydrostatic pressure, which have had significant positive effects on improving quality, increasing shelf life, and ensuring safety in other meats, could be promising options for future development. The application of these technologies, along with considering consumer acceptance and market requirements, could provide a new path for sustainable exploitation and wider commercialization of camel meat. Given the increasing global interest in camel meat as a healthy and affordable protein source, the investigation and development of its preservation methods is of increasing importance. A review of past studies shows that a wide range of technologies have been used or investigated to increase the shelf life and safety of camel meat; from traditional methods such as salting, drying, and smoking to modern methods such as chilling, freezing, and vacuum packaging. Also, the use of additives and preservatives, both natural (herbal, essential oils) and synthetic (nitrates, sulfites), has been reported to be effective in controlling microbial growth and delaying spoilage of camel meat.

## 14 Limitations and Future Perspectives

Recent advances in MAP, new technologies such as irradiation, nanotechnology, as well as biological methods including the use of beneficial microorganisms and antimicrobial peptides, have opened new horizons for increasing the shelf life and safety of camel meat. On the other hand, consumer acceptance and market trends towards healthy, natural and minimally processed products are also guiding the development of these technologies. However, many of these technologies have not yet been directly evaluated on camel meat. In particular, super‐chilling, which has been shown to effectively reduce microbial growth and oxidation in beef, poultry, and fish, and high hydrostatic pressure, which has been successful in inactivating pathogenic microorganisms in other meats, have yet to be studied in the context of camel meat. Given the unique characteristics of camel meat (low intramuscular fat, high moisture, and specific protein structure), the successful application of these technologies can not only improve the safety and shelf life of camel meat, but also facilitate its acceptance in international markets as a healthy, innovative, and high‐quality red meat.

While this review provides a comprehensive overview of preservation strategies for camel meat, it has certain limitations. The primary limitation is the scarcity of published studies specifically focusing on emerging non‐thermal technologies (such as high‐pressure processing and pulsed electric fields) applied to camel meat, compared to beef or poultry. Most available data are extrapolated from other meat sources. Furthermore, while laboratory‐scale results for natural preservatives are promising, their economic feasibility and regulatory acceptance for industrial‐scale application in camel meat processing remain under‐researched. Future studies should focus on validating these novel technologies directly on camel meat and assessing consumer acceptance of processed camel products in global markets.

## Author Contributions

**Najmeh Rezaei:** conceptualization, writing – original draft, writing – review and editing. **Mohammad Ali Hesarinejad:** conceptualization, writing – original draft, writing – review and editing, project administration. **Farnaz Rezaiyan Attar:** conceptualization, writing – original draft, writing – review and editing. **Nasser Morgan Azghadi:** conceptualization, writing – review and editing, writing – original draft.

## Funding

The authors have nothing to report.

## Ethics Statement

The authors have nothing to report.

## Consent

All authors have read and agreed to the published version of the manuscript. All authors have read and approved the final manuscript.

## Conflicts of Interest

The authors declare no conflicts of interest.

## Data Availability

Data sharing not applicable to this article as no datasets were generated or analyzed during the current study.

## References

[fsn371730-bib-0001] Abd Elgadir, M. , A. A. Mariod , N. Alrumaih , S. H. S. Mohamed , M. A. Aladhadh , and R. R. Alayouni . 2025. “Quality Evaluation of Fresh Camel Meat Dipped in Edible Citric Acid.” Theory and Practice of Meat Processing 84: 84–90.

[fsn371730-bib-0002] Al‐Ani, F. , and J. Roberson . 2005. “Fungal Infection of Camelids.” In Desertification Combat and Food Safety‐The Added Value of Camel Producers, 70–84. IOS Press.

[fsn371730-bib-0003] Al‐Bachir, M. , and R. Zeinou . 2009. “Effect of Gamma Irradiation on Microbial Load and Quality Characteristics of Minced Camel Meat.” Meat Science 82, no. 1: 119–124. 20416783 10.1016/j.meatsci.2008.12.012

[fsn371730-bib-0004] Al‐Delaimy, K. H. S. , and M. M. F. Barakat . 1971. “Antimicrobial and Preservative Activity of Garlic on Fresh Ground Camel Meat: I.—Effect of Fresh Ground Garlic Segments.” Journal of the Science of Food and Agriculture 22, no. 2: 96–98. 5102504 10.1002/jsfa.2740220214

[fsn371730-bib-0005] Aljabeili, H. S. , E. A. Abd El‐Hady , M. M. Abd El‐Razik , and M. Abd Elgadir . 2016. “Quality Characteristics of Cured Semi Dried Camel Meat Treated With Different Medicinal Plants as Natural Preservatives Sources.” Journal of Agricultural and Veterinary Sciences 9, no. 1: 81–91.

[fsn371730-bib-0006] Al‐Juhaimi, F. Y. , I. A. Mohamed Ahmed , O. Q. Adiamo , et al. 2018. “Effect of Argel (*Solenostemma argel*) Leaf Powder on the Quality Attributes of Camel Patties During Cold Storage.” Journal of Food Processing and Preservation 42, no. 2: e13496.

[fsn371730-bib-0007] Alla, D. 2008. “The Effects of Preservation Periods on Meat Characteristics of Camel and Cattle.” Research Journal of Biological Sciences 3, no. 6: 616–619.

[fsn371730-bib-0008] Ansarian, E. , M. Aminzare , H. H. Azar , M. R. Mehrasbi , and M. Bimakr . 2022. “Nanoemulsion‐Based Basil Seed Gum Edible Film Containing Resveratrol and Clove Essential Oil: In Vitro Antioxidant Properties and Its Effect on Oxidative Stability and Sensory Characteristic of Camel Meat During Refrigeration Storage.” Meat Science 185: 108716. 34839195 10.1016/j.meatsci.2021.108716

[fsn371730-bib-0009] Asif, A. , F. Ibrahim , and A. Ansari . 2024. “A Systematic Review: Effectiveness of Herbs and Spices as Natural Preservatives to Enhance Meat Shelf‐Life: Herbs and Spices as Natural Preservatives.” Journal of Health and Rehabilitation Research 4, no. 3: 1–7.

[fsn371730-bib-0010] Atika, B. , A. Abdelkadder , and B. Baaissa . 2014. “Microbiological Characterization of Camel and Sheep Meat Preserved by Refrigeration and Lactic Acid.” Emirates Journal of Food and Agriculture 26, no. 5: 465.

[fsn371730-bib-0011] Baba, W. N. , N. Rasool , M. Selvamuthukumara , and S. Maqsood . 2021. “A Review on Nutritional Composition, Health Benefits, and Technological Interventions for Improving Consumer Acceptability of Camel Meat: An Ethnic Food of Middle East.” Journal of Ethnic Foods 8, no. 1: 18.

[fsn371730-bib-0012] Babiker, S. A. , and O. K. Yousif . 1990. “Chemical Composition and Quality of Camel Meat.” Meat Science 27, no. 4: 283–287. 22055364 10.1016/0309-1740(90)90066-F

[fsn371730-bib-0084] Ben Braïek, O. , and S. Smaoui 2021. “Chemistry, safety, and challenges of the use of organic acids and their derivative salts in meat preservation.” Journal of Food Quality 2021, no. 1: 6653190.

[fsn371730-bib-0014] Benyagoub, E. , and C. Bessadet . 2023. “A Survey on Dried and Salted Camel Meat (Kadid): A Traditional Meat By‐Product of Southern Algeria.” International Journal on Nutraceuticals Functional Foods and Novel Foods From Research to Industrial Applications 2: 528–536.

[fsn371730-bib-0013] Benyagoub, E. , M. Kerkoub , and C. Bessadet . 2025. “An Investigation Into the Traditional Processing of Camel Meat as Khlii: A Dried, Cooked and Fatty Meat From Southwestern Algeria.” Food Research 1, no. 7: 7.

[fsn371730-bib-0015] Bhagath, Y. B. , and K. Manjula . 2019. “Influence of Composite Edible Coating Systems on Preservation of Fresh Meat Cuts and Products: A Brief Review on Their Trends and Applications.” International Food Research Journal 26, no. 2: 377–392.

[fsn371730-bib-0016] Busboom, J. R. 2003. “Curing and Smoking Poultry Meat.” Issued by Washington State Cooperative Extension and the U. S. Department of Agriculture. Subject code 665. A. EB1660.

[fsn371730-bib-0017] Carballo, D. E. , J. Mateo , S. Andrés , et al. 2019. “Microbial Growth and Biogenic Amine Production in a Balkan‐Style Fresh Sausage During Refrigerated Storage Under a CO_2_‐Containing Anaerobic Atmosphere: Effect of the Addition of Zataria Multiflora Essential Oil and Hops Extract.” Antibiotics 8, no. 4: 227. 31731685 10.3390/antibiotics8040227 PMC6963869

[fsn371730-bib-0018] Chellaiah, R. , M. Shanmugasundaram , and J. Kizhekkedath . 2020. “Advances in Meat Preservation and Safety.” International Journal of Science and Research 9, no. 3: 1499–1502.

[fsn371730-bib-0019] Djenane, D. , M. Aboudaou , F. Djenane , D. García‐Gonzalo , and R. Pagán . 2020. “Improvement of the Shelf‐Life Status of Modified Atmosphere Packaged Camel Meat Using Nisin and *Olea europaea* Subsp. *laperrinei* Leaf Extract.” Food 9, no. 9: 1336. 10.3390/foods9091336 PMC7555406 32971898

[fsn371730-bib-0020] Djenane, D. , B. M. Khaled , Y. Ben Miri , et al. 2024. “Improved Functionality, Quality, and Shelf Life of Merguez‐Type Camel Sausage Fortified With Spirulina as a Natural Ingredient.” Food 14, no. 1: 59. 10.3390/foods14010059 PMC11719629 39796348

[fsn371730-bib-0021] Ebrahimian, M. , and M. Mohsenzadeh . 2023. “Antimicrobial and Antioxidant Effects of Chitosan Film Containing Nanoemulsion of *Melissa officinalis* L. Extract and Bunium Persicum Essential Oil on *Listeria monocytogenes* Inoculated Into Camel Meat.” Journal of Food Science and Technology (Iran) 19, no. 133: 249–264.

[fsn371730-bib-0071] Edris, E. A. M. , F. A. Shaltout , and H. M. Lamada . 2020. “Bacteriological Examination of Some Ready to Eat Meat and Chicken Meals.” Benha Veterinary Medical Journal 38, no. 2: 76–79.

[fsn371730-bib-0022] El‐Faer, M. Z. , T. N. Rawdah , K. M. Attar , and M. V. Dawson . 1991. “Mineral and Proximate Composition of the Meat of the One‐Humped Camel ( *Camelus dromedarius* ).” Food Chemistry 42, no. 2: 139–143.

[fsn371730-bib-0023] Elgasim, E. A. , and M. A. Alkanhal . 1992. “Proximate Composition, Amino Acids and Inorganic Mineral Content of Arabian Camel Meat: Comparative Study.” Food Chemistry 45, no. 1: 1–4.

[fsn371730-bib-0024] Elsharawy, N. T. , A. A. ALtowairqi , W. A. Alshehri , and A. M. Baghdadi . 2026. “Antimicrobial and Shelf‐Life Extension Effects of Arabic Gum as a Natural Preservative for Camel Meat.” Egyptian Journal of Veterinary Science 57, no. 1: 315–325.

[fsn371730-bib-0025] Engy, F. , and F. Mohamed . 2018. “Effect of Adding Leek as a Natural Source of Nitrite on the Processing and Quality Properties of Camel Sausage During Frozen Storage.” Journal of Advances in Food Science & Technology 5, no. 1: 27–33.

[fsn371730-bib-0026] Eskandari, M. H. , M. Majlesi , H. R. Gheisari , A. Farahnaky , and Z. Khaksar . 2013. “Comparison of Some Physicochemical Properties and Toughness of Camel Meat and Beef.” Journal of Applied Animal Research 41, no. 4: 442–447.

[fsn371730-bib-0027] Fallah, A. A. , H. Tajik , and A. A. Farshid . 2010. “Chemical Quality, Sensory Attributes and Ultrastructural Changes of Gamma‐Irradiated Camel Meat.” Journal of Muscle Foods 21, no. 3: 597–613.

[fsn371730-bib-0028] Feldhusen, F. , T. Kirschner , R. Koch , W. Giese , and S. Wenzel . 1995. “Influence on Meat Colour of Spray‐Chilling the Surface of Pig Carcasses.” Meat Science 40, no. 2: 245–251. 22059976 10.1016/0309-1740(94)00047-b

[fsn371730-bib-0029] Fista, G. A. , J. G. Bloukas , and A. S. Siomos . 2004. “Effect of Leek and Onion on Processing and Quality Characteristics of Greek Traditional Sausages.” Meat Science 68, no. 2: 163–172. 22062225 10.1016/j.meatsci.2004.02.005

[fsn371730-bib-0030] Gheisari, H. R. , and M. Eskandari . 2013. “Effect of Curing on Camel Meat Lipid Oxidation and Enzymatic Activity During Refrigerated Storage.” VETERINARSKI ARHIV 83, no. 5: 551–562.

[fsn371730-bib-0031] Hamad, A. , and S. Abdullah . 2025. “Evaluation of Some Nutritional Properties of Camel Meat Originating From Libya.” Al‐Qalam Journal of Medical and Applied Sciences 8: 15–19.

[fsn371730-bib-0032] Hamrouni, I. , O. Abdelhedi , N. Zouari , N. Fakhfekh , and M. Jridi . 2023. “Ultrasound‐Assisted Enzymatic Hydrolysis of Camel Skin Gelatin: Application of Hydrolysates as Bioactive in Edible Coatings to Prolong Camel Meat Shelf‐Life.” SSRN 5216446.

[fsn371730-bib-0033] Hansen, E. , D. Juncher , P. Henckel , A. Karlsson , G. Bertelsen , and L. H. Skibsted . 2004. “Oxidative Stability of Chilled Pork Chops Following Long Term Freeze Storage.” Meat Science 68, no. 3: 479–484. 22062417 10.1016/j.meatsci.2004.05.002

[fsn371730-bib-0034] Hassanien, M. , T. El‐Khateib , M. Hassan , and A. M. Abd‐El‐Malek . 2022. “Changes in Camel and Cattle Meat During Chilling Preservation.” Assiut Veterinary Medical Journal 68, no. 173: 1–9.

[fsn371730-bib-0035] Hussein, A. M. , W. R. El‐Ghareeb , and O. O. Lotfy . 2012. “Shelf Life Improvement of Camel Meat Treated With Potassium Sorbate 0.3%.” Journal of American Science 8, no. 4: 507–511.

[fsn371730-bib-0036] Hussein, M. A. , S. H. Mohamed , A. E.‐S. Hafez , et al. 2021. “Impact of Natural and Chemical Agents on Quality and Biogenic Amine Formation of Chilled Camel Minced Meat.” Slovenian Veterinary Research 58: 71–82.

[fsn371730-bib-0037] Jenkins, W. A. , and J. P. Harrington . 1991. “Packaging of Foods With Plastics.” Technomic Publishing Co.

[fsn371730-bib-0038] Jiang, J. , and Y. L. Xiong . 2016. “Natural Antioxidants as Food and Feed Additives to Promote Health Benefits and Quality of Meat Products: A Review.” Meat Science 120: 107–117. 27091079 10.1016/j.meatsci.2016.04.005

[fsn371730-bib-0039] Jouki, M. , and N. Khazaei . 2012. “Lipid Oxidation and Color Changes of Fresh Camel Meat Stored Under Different Atmosphere Packaging Systems.” Journal of Food Processing & Technology 3, no. 11: 1000189.

[fsn371730-bib-0040] Jumaa, W. F. H. F. F. n.d. “Effect of Peppermint Oil Extract on the Quality Characteristics of Cold‐Preserved Camel Meat.”

[fsn371730-bib-0042] Kadim, I. T. , I. S. Al‐Amri , A. Y. Alkindi , and Q. M. I. Haq . 2022. “Nutritional Values and Health Benefits of Dromedary Camel Meat.” Animal Frontiers 12, no. 4: 61–70. 10.1093/af/vfac051 PMC9374515 35974788

[fsn371730-bib-0041] Kadim, I. T. , O. Mahgoub , W. Al‐Marzooqi , S. Al‐Zadjali , K. Annamalai , and M. H. Mansour . 2006. “Effects of Age on Composition and Quality of Muscle *Longissimus thoracis* of the Omani Arabian Camel (*Camelus dromedaries*).” Meat Science 73, no. 4: 619–625. 22062561 10.1016/j.meatsci.2006.03.002

[fsn371730-bib-0043] Kadim, I. T. , O. Mahgoub , and M. Mbaga . 2014. “Potential of Camel Meat as a Non‐Traditional High Quality Source of Protein for Human Consumption.” Animal Frontiers 4, no. 4: 13–17.

[fsn371730-bib-0044] Kalalou, I. , M. Faid , and T. A. Ahami . 2004. “Improving the Quality of Fermented Camel Sausage by Controlling Undesirable Microorganisms With Selected Lactic Acid Bacteria.” International Journal of Agriculture and Biology 6: 447–451.

[fsn371730-bib-0045] Kalalou, I. , I. Zerdani , and M. Faid . 2010. “Antagonistic Action of Biopreservative *Lactobacillus plantarum* Strain on Pathogenic *E. coli* O157: H7 in Fresh Camel Meat Stored at 10°C.” World Journal of Dairy & Food Sciences 5, no. 1: 07–13.

[fsn371730-bib-0046] Kamel, S. M. 2013. “The Use of Some Herbs for Improving the Refrigerated Storage Stability of Minced Camel Meat.” Scientific Journal of Microbiology 2: 95–103.

[fsn371730-bib-0047] Kerry, J. P. , M. N. O'grady , and S. A. Hogan . 2006. “Past, Current and Potential Utilisation of Active and Intelligent Packaging Systems for Meat and Muscle‐Based Products: A Review.” Meat Science 74, no. 1: 113–130. 22062721 10.1016/j.meatsci.2006.04.024

[fsn371730-bib-0048] Khezrian, A. , and Y. Shahbazi . 2018. “Application of Nanocompostie Chitosan and Carboxymethyl Cellulose Films Containing Natural Preservative Compounds in Minced Camel's Meat.” International Journal of Biological Macromolecules 106: 1146–1158. 28847602 10.1016/j.ijbiomac.2017.08.117

[fsn371730-bib-0049] Lambert, A. D. , J. P. Smith , and K. L. Dodds . 1991. “Shelf Life Extension and Microbiological Safety of Fresh Meat—A Review.” Food Microbiology 8, no. 4: 267–297.

[fsn371730-bib-0050] Lawrie, R. A. , and D. A. Ledward . 2006. Lawrie's Meat Science. Woodhead Publishing.

[fsn371730-bib-0051] Manheem, K. , O. Adiamo , U. Roobab , et al. 2023. “A Comparative Study on Changes in Protein, Lipid and Meat‐Quality Attributes of Camel Meat, Beef and Sheep Meat (Mutton) During Refrigerated Storage.” Animals 13, no. 5: 904. 36899761 10.3390/ani13050904 PMC10000245

[fsn371730-bib-0052] Maqsood, S. , A. Abushelaibi , K. Manheem , A. Al Rashedi , and I. T. Kadim . 2015. “Lipid Oxidation, Protein Degradation, Microbial and Sensorial Quality of Camel Meat as Influenced by Phenolic Compounds.” LWT‐ Food Science and Technology 63, no. 2: 953–959.

[fsn371730-bib-0053] Maqsood, S. , A. Abushelaibi , K. Manheem , and I. T. Kadim . 2015. “Characterisation of the Lipid and Protein Fraction of Fresh Camel Meat and the Associated Changes During Refrigerated Storage.” Journal of Food Composition and Analysis 41: 212–220.

[fsn371730-bib-0054] Maqsood, S. , N. A. Al Haddad , and P. Mudgil . 2016. “Vacuum Packaging as an Effective Strategy to Retard Off‐Odour Development, Microbial Spoilage, Protein Degradation and Retain Sensory Quality of Camel Meat.” LWT‐ Food Science and Technology 72: 55–62.

[fsn371730-bib-0055] McCance, R. A. , and E. M. Widdowson . 2014. McCance and Widdowson's the Composition of Foods. Royal Society of Chemistry.

[fsn371730-bib-0057] McMillin, K. W. 2008. “Where Is MAP Going? A Review and Future Potential of Modified Atmosphere Packaging for Meat.” Meat Science 80, no. 1: 43–65. 22063169 10.1016/j.meatsci.2008.05.028

[fsn371730-bib-0056] McMillin, K. W. , N. Y. Huang , C. P. Ho , and B. S. Smith . 1999. “Quality and Shelf‐Life of Meat in Case‐Ready Modified Atmosphere Packaging.” In Quality Attributes of Muscle Foods, 73–93. Springer.

[fsn371730-bib-0058] Millette, M. , F.‐M. Luquet , and M. Lacroix . 2007. “In Vitro Growth Control of Selected Pathogens by *Lactobacillus acidophilus* ‐and *Lactobacillus casei* ‐Fermented Milk.” Letters in Applied Microbiology 44, no. 3: 314–319. 17309510 10.1111/j.1472-765X.2006.02060.x

[fsn371730-bib-0059] Moghimi, N. , A. Khanjari , A. Misaghi , et al. 2021. “Extending the Shelf Life of Fresh Camel Sausage by the Integration of *Cuminum cyminum* L. Essential Oil.” Journal of Nutrition, Fasting & Health 9, no. 4: 334–341.

[fsn371730-bib-0060] Murbach Teles Andrade, B. F. , L. Nunes Barbosa , I. da Silva Probst , and A. Fernandes Júnior . 2014. “Antimicrobial Activity of Essential Oils.” Journal of Essential Oil Research 26, no. 1: 34–40.

[fsn371730-bib-0061] Odeyemi, O. A. , O. O. Alegbeleye , M. Strateva , and D. Stratev . 2020. “Understanding Spoilage Microbial Community and Spoilage Mechanisms in Foods of Animal Origin.” Comprehensive Reviews in Food Science and Food Safety 19, no. 2: 311–331. 33325162 10.1111/1541-4337.12526

[fsn371730-bib-0062] Osaili, T. M. , F. Hasan , A. A. Al‐Nabulsi , et al. 2021. “Effect of Essential Oils and Vacuum Packaging on Spoilage‐Causing Microorganisms of Marinated Camel Meat During Storage.” Food 10, no. 12: 2980. 10.3390/foods10122980 PMC8701313 34945531

[fsn371730-bib-0063] Peng, Y. , and Y. Li . 2014. “Combined Effects of Two Kinds of Essential Oils on Physical, Mechanical and Structural Properties of Chitosan Films.” Food Hydrocolloids 36: 287–293.

[fsn371730-bib-0064] Preedy, V. R. 2015. Essential Oils in Food Preservation, Flavor and Safety. Academic Press.

[fsn371730-bib-0065] Raiymbek, G. , I. T. Kadim , O. Mahgoub , G. Konuspayeva , and B. Faye . 2025. “Comparative Minerals and Vitamins Composition of Bactrian ( *Camelus bactrianus* ) and Dromedary ( *Camelus dromedarius* ) Meat.” Food Science and Engineering 6, no. 2: 226–243.

[fsn371730-bib-0066] Reza Gheisari, H. , M. Aminlari , and S. Shahram Shekarforoush . 2009. “A Comparative Study of the Biochemical and Functional Properties of Camel and Cattle Meat During Frozen Storage.” Veterinarski Arhiv 79, no. 1: 51–68.

[fsn371730-bib-0067] Shabani, M. , A. Ghorbani‐HasanSaraei , N. Shariatifar , F. Savadkoohi , and S.‐A. Shahidi . 2023. “Effect of *Urtica dioica* L. Essential Oil (Forms of Free and Nanoliposome) on Some Inoculated Pathogens (*Escherichia coli* and *Listeria monocytogenes* ) in Minced Camel Meat.” Food Chemistry: X 20: 101050. 38144767 10.1016/j.fochx.2023.101050 PMC10740059

[fsn371730-bib-0068] Shahbazi, Y. , N. Karami , and N. Shavisi . 2018. “Effect of *Mentha spicata* Essential Oil on Chemical, Microbial, and Sensory Properties of Minced Camel Meat During Refrigerated Storage.” Journal of Food Safety 38, no. 1: e12375.

[fsn371730-bib-0069] Shaltout, F. A. , A. A. Ahmed , E. Maarouf , and M. K. Ahmed . 2018. “Heavy Metal Residues in Chicken Cuts Up and Processed Chicken Meat Products.” Benha Veterinary Medical Journal 34, no. 1: 473–483.

[fsn371730-bib-0070] Shaltout, F. A. , E. M. El‐diasty , M. Elmesalamy , and M. Elshaer . 2014. “Study on Fungal Contamination of Some Chicken Meat Products With Special Reference to 2 the Use of PCR for Its Identification. Conference.” Veterinary Medical Journal 60: 1–10.

[fsn371730-bib-0072] Siragusa, G. R. , and J. S. Dickson . 1992. “Inhibition of *Listeria monocytogenes* on Beef Tissue by Application of Organic Acids Immobilized in a Calcium Alginate Gel.” Journal of Food Science 57, no. 2: 293–296.

[fsn371730-bib-0073] Sobhy, A. , and F. Shaltout . 2020. “Prevalence of Some Food Poisoning Bacteria in Semi Cooked Chicken Meat Products at Kaliobyia Governorate With Using of Recent Vitek 2 Compact and PCR Techniques.” Benha Veterinary Medical Journal 38, no. 2: 88–92.

[fsn371730-bib-0074] Tajkarimi, M. M. , S. A. Ibrahim , and D. O. Cliver . 2010. “Antimicrobial Herb and Spice Compounds in Food.” Food Control 21, no. 9: 1199–1218.

[fsn371730-bib-0075] Takahashi, K. 1996. “Structural Weakening of Skeletal Muscle Tissue During Post‐Mortem Ageing of Meat: The Non‐Enzymatic Mechanism of Meat Tenderization.” Meat Science 43: 67–80. 10.1016/0309-1740(96)00056-3 22060642

[fsn371730-bib-0076] Tang, H. , W. S. Darwish , W. R. El‐Ghareeb , et al. 2020. “Microbial Quality and Formation of Biogenic Amines in the Meat and Edible Offal of Camelus Dromedaries With a Protection Trial Using Gingerol and Nisin.” Food Science & Nutrition 8, no. 4: 2094–2101. 32328276 10.1002/fsn3.1503 PMC7174210

[fsn371730-bib-0077] Tegegne, H. A. , A. Berhanu , Y. Getachew , et al. 2019. “Microbiological Safety and Hygienic Quality of Camel Meat at Abattoir and Retail Houses in Jigjiga City, Ethiopia.” Journal of Infection in Developing Countries 13, no. 3: 188–194. 32040447 10.3855/jidc.9686

[fsn371730-bib-0078] Teshome, E. , S. F. Forsido , H. P. V. Rupasinghe , and E. Olika Keyata . 2022. “Potentials of Natural Preservatives to Enhance Food Safety and Shelf Life: A Review.” Scientific World Journal 2022, no. 1: 9901018. 36193042 10.1155/2022/9901018 PMC9525789

[fsn371730-bib-0079] Wambui, J. M. , P. O. Lamuka , and P. M. K. Njage . 2017. “Lactic Acid Bacteria Isolates From Fermented Camel Milk (Suusac) Are Potential Protective Cultures of Raw Camel Meat.” International Journal of Agriculture and Environmental Research 3, no. 3: 2960–2975.

[fsn371730-bib-0080] Yehia, H. M. , A. H. Al‐Masoud , M. F. Elkhadragy , et al. 2021. “Improving the Quality and Safety of Fresh Camel Meat Contaminated With *Campylobacter jejuni* Using Citrox, Chitosan, and Vacuum Packaging to Extend Shelf Life.” Animals 11, no. 4: 1152. 33920579 10.3390/ani11041152 PMC8072804

[fsn371730-bib-0081] Zaki, E. F. 2017. “The Quality Characteristics of Camel Sausage Formulated With Different Levels of Whey Protein Powder.” International Journal of Environment, Agriculture and Biotechnology 2, no. 5: 238929.

[fsn371730-bib-0082] Zegeye, A. 1999. “A Note on the Influence of Heat Treatment, Salting and Smoking on the Acceptability of Camel Meat Products.” Meat Science 53, no. 4: 217–219. 22063462 10.1016/s0309-1740(99)00057-1

[fsn371730-bib-0083] Zhou, G. H. , X. L. Xu , and Y. Liu . 2010. “Preservation Technologies for Fresh Meat–A Review.” Meat Science 86, no. 1: 119–128. 20605688 10.1016/j.meatsci.2010.04.033

